# Expecting, understanding, relating, and interacting-older, middle-aged and younger adults’ perspectives on breakdown situations in human–robot dialogues

**DOI:** 10.3389/frobt.2022.956709

**Published:** 2022-10-14

**Authors:** Maitreyee Tewari, Helena Lindgren

**Affiliations:** Umeå University, Umeå, Sweden

**Keywords:** Activity Theory, human–robot interaction, breakdown situations, focus shift, qualitative study, social robotics, argumentation theory

## Abstract

**Purpose:** The purpose of this study is to explore how older, middle aged and younger adults perceive breakdown situations caused by lack of or inconsistent knowledge, sudden focus shifts, and conflicting intentions in dialogues between a human and a socially intelligent robot in a home environment, and how they perceive strategies to manage breakdown situations.

**Methods:** Scenarios embedding dialogues on health-related topics were constructed based on activity-theoretical and argumentation frameworks. Different reasons for breakdown situations and strategies to handle these were embedded. The scenarios were recorded in a Wizard-of-Oz setup, with a human actor and a Nao robot. Twenty participants between 23 and 72 years of age viewed the recordings and participated in semi-structured interviews conducted remotely. Data were analyzed qualitatively using thematic analysis.

**Results:** Four themes relating to breakdown situations emerged: *expecting*, *understanding*, *relating*, and *interacting*. The themes span complex human activity at different complementary levels and provide further developed understanding of breakdown situations in human–robot interaction (HRI). Older and middle-aged adults emphasized emphatic behavior and adherence to social norms, while younger adults focused on functional aspects such as gaze, response time, and length of utterances. A hierarchical taxonomy of aspects relating to breakdown situations was formed, and design implications are provided, guiding future research.

**Conclusion:** We conclude that a socially intelligent robot agent needs strategies to 1) construct and manage its understanding related to emotions of the human, social norms, knowledge, and motive on a higher level of meaningful human activity, 2) act accordingly, for instance, adhering to transparent social roles, and 3) resolve conflicting motives, and identify reasons to prevent and manage breakdown situations at different levels of collaborative activity. Furthermore, the novel methodology to frame the dynamics of human–robot dialogues in complex activities using Activity Theory and argumentation theory was instrumental in this work and will guide the future work on tailoring the robot’s behavior.

## 1 Introduction

Humans tend to attribute human-like characteristics such as beliefs and intentions to human-like robots such as the Pepper and Nao robots^1^, a phenomenon denoted as anthropomorphism Waytz et al. (2010). This leads to expectations from robots to act in specific ways in social situations, such as when a person has a conversation about health in a care situation, for instance, as a part of a healthcare intervention in the person’s home. Similar to dialogues between humans, situations of problems in understanding will occur for different reasons in dialogues between humans and social robots, in which the robot and the human need to manage as the conversation unfolds. The research presented in this article aims to study how older, middle-aged and younger adults experience situations denoted in this work *breakdown situations*, caused by lack of knowledge, sudden focus shifts, or conflicting motives in dialogues between the human and a service robot in a home environment.

Researchers have studied how people experience natural dialogues about health topics in care situations between humans and embodied virtual agents. The studies show that empathetic behavior is expected, has a positive impact, and gives the users positive experiences from interacting with the agent (Stal ter et al., 2020). Empathy is defined by Cuff et al. (2016) as “The act of perceiving, understanding, experiencing, and responding to another person’s emotional state and ideas.” For a socially intelligent software agent to act with empathy, it requires a model of the human, sometimes denoted Theory of Mind (ToM) Rabinowitz et al. (2018); Cuzzolin et al. (2020); Çelikok et al. (2019), as well as a model of the situation, representing a part of the agent’s knowledge and understanding. Such knowledge and understanding should also include strategies for managing situations when knowledge and understanding, or lack thereof, may cause breakdown situations.

A breakdown situation is typically treated as an error when evaluating robots’ capability to interact with people Marge and Rudnicky (2019); Schütte et al. (2017). By contrast, breakdown situations are considered a natural element of a learning and development process from an activity-theoretical perspective (Bødker and Andersen, 2005). Adopting this alternative perspective could help shift the focus from failure to opportunities to learn something new. This research is based on such an interpretation of breakdown situations to explore how younger and older adults perceive breakdown situations and the robot’s ways of managing them.

So far, research on human–robot interaction (HRI) has focused on developing methods to recognize and enact emotions (Tian and Oviatt, 2021), roles (Deng et al., 2019; Lee et al., 2012; Epley et al., 2007), and relationships (Breazeal and Scassellati, 1999) to increase social capability in robots and to provide a positive experience for humans interacting with the robots. However, recent research shows that understanding breakdown situations and their management are equally significant for building relatedness and naturalness in HRI (Tian and Oviatt, 2021; Ragni et al., 2016; Mirnig et al., 2017; Lee et al., 2010; Salem et al., 2015). Even though some work has been carried out on integrating breakdown situations for goal-driven interactions (Schütte et al., 2017; Marge and Rudnicky, 2019; Sklar and Azhar, 2015), there is a lack of studies that investigate how people experience situations where humans and robots manage miscommunications, lack of understanding, and other reasons for breakdown situations in dialogues that embed both goal-driven content and those with less clear purposes. To address such dialogues, we apply the activity-theoretical framework for understanding meaningful human activity as a continuous development process and focus on dialogues between a human and socially intelligent robot in a home care situation. The following research questions are addressed:

1) (RQ1) What aspects do people perceive relating to breakdown situations in a human–robot dialogue?
2) (RQ2) Are there differences between older and younger participants’ perception of breakdown situations and of the robot’s behavior?
3) (RQ3) How do the participants expect the robot to manage breakdown situations?
4) (RQ4) How does anthropomorphism relate to participants’ experience of human–robot dialogues?

The research questions are addressed through a user study.

The contributions of this work are the following: 1) further developed an understanding of how Older, middle aged and younger people people perceive breakdown situations in human–robot dialogues on health-related topics; 2) a novel Activity Theory-based methodology for designing and understanding dialogues between humans and robots as a part of purposeful human activity; 3) a hierarchical taxonomy of perceived aspects contributing to breakdown situations in HRI scenarios, and 4) a list of design implications emerging from participant’s reported expectations for embedding empathetic capability in socially intelligent agents.

The article is organized as follows: a background on socially intelligent agents embedded in robots is provided in the following section. Activity Theory and related research on breakdown situations for HRI are introduced. In Section 3 the methodology is presented, and the results are provided in Section 4. The methodology and the results are discussed in Section 5, and Section 6 concludes the article.

## 2 Background and related work

In this section, definitions of social robots are introduced in order to provide perspective on a socially capable robot applied in our study. Furthermore, activity-theoretical concepts used in this work are introduced and a few examples of applications of Activity Theory so far in HRI research. Related work on breakdown situations in HRI is also summarized.

### 2.1 Socially intelligent robots and human–robot interactions

Social aspects relating to humans interacting with robots have been studied from the perspective of human social intelligence to create robot behavior, which is intuitive and relevant for interacting with humans (Dautenhahn, 2007). Interactions in social situations involving humans and robots require understanding and exchanging social cues in the form of verbal and non-verbal communication Onyeulo and Gandhi (2020), yet what we still have is “the simple command only” type of communication as discussed by Mavridis (2015). To transition from command-driven to a more natural day-to-day social interaction requires a socially intelligent robot that acts within the bounds of its role, expected behavior, and the goals that guide its actions. Such social robots have been defined in the following ways:

1) Socially evocative and sociable—“that rely on the human tendency to anthropomorphize; proactively engage with humans to fulfill internal aims” (Dautenhahn,2007). Such robots can interact with humans and other agents in a socially acceptable and interpretable manner to convey their beliefs and perceptions about the world to fulfill their intentions Breazeal (2002); Breazeal (2003); Breazeal and Scassellati (1999).
2) Socially intelligent—“robots that show aspects of human social cognition requiring deep models of human intelligence with social competence” Dautenhahn (1998).
3) Socially situated, socially interactive—“robots situated in a social environment with the capability of distinguishing other social agents, or robots for which social interaction plays a key role.” Such robots can recognize and display emotions, have a distinctive personality, manage uncertainty, use dialogues and gestures for communication, and can initiate and maintain relationships Fong et al. (2003).

A *Socially Intelligent Robot* as applied in this study spans these definitions since participants’ expectations are investigated and is in addition, interpreted as a *we-intentional* actor following the work of Tuomela (2005). By we-intention, it is assumed that the robot strives to maintain a joint intention about the activity with the human actor. This is key in the collaborative health-related activities in a home environment focused in this work. The robot also has roles and rules to follow and uses tools (physical or perceptual), conforming to social and conversational norms on how the activity is expected to be conducted within the community of actors. The subsequent section introduces such activities from an activity-theoretical perspective.

### 2.2 Introduction to Activity Theory

Activity Theory originated in the work of Vygotsky (1978) and Leontiev (1978), providing models of human activity and development. The models have been further developed when applied to research areas such as development work research (Engeström, 1987; Engeström, 1995; Engeström, 1999b), human–computer interaction (HCI) (Bødker and Andersen, 2005; Kaptelinin and Nardi, 2006), and computer-supported cooperative work (CSCW) (Bardram, 1997; Engeström, 1999a).

**FIGURE 1:**
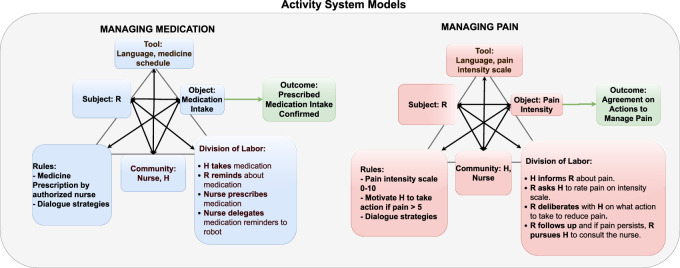
Example of activity system models interpreted using the Engeström’s model for the activities managing medications and managing pain. The activity systems provide a template for instantiating the activity, including the knowledge required about actors, objects, rules, community, tools, division of labor, and outcome from a robot’s perspective is exemplified. R = Companion Robot and H = Human.

From an activity-theoretical perspective, purposeful *activity* is defined by its *object*, toward which the *subject* performing the activity is directing his/her actions (Leontiev, 1978). The activity has a *motive* fulfilling an individual’s need. Central to activity are *mediating tools*, which the subject uses to affect and transform the object of the activity. This interplay between the three core components of human activity: subject, object, and tool are often visualized as a triangle, exemplified by the upper part of the triangles in Figure 1 (Leontiev, 1978).

Engeström (1999a) extended the model to capture more elements of the organizational context commonly affecting human activity in work situations. The extended model captures collectives of subjects in collaborative activity, including the elements *division of labor*, *community/team*, and *rules* governing the activity. In our scenario, the activity system model embeds social and cultural factors of the community formed by actors. In addition to the person in focus and their social network, including healthcare personnel, we include the robot as an actor driven by motives adhering to regulations. The community members follow *rules* such as norms, conventions, and prescriptions for an activity, such as managing medication and *division of labor*, directing how the actors organize their actions within the collective activity. The *outcome* of the collective activity results from transformations performed on the object by the activity in question. Examples from the robot’s perspective as the subject in the activity system models are provided in Figure 1. In our example, the robot partners with a team consisting of the person being cared for and the nurses.

**FIGURE 2:**
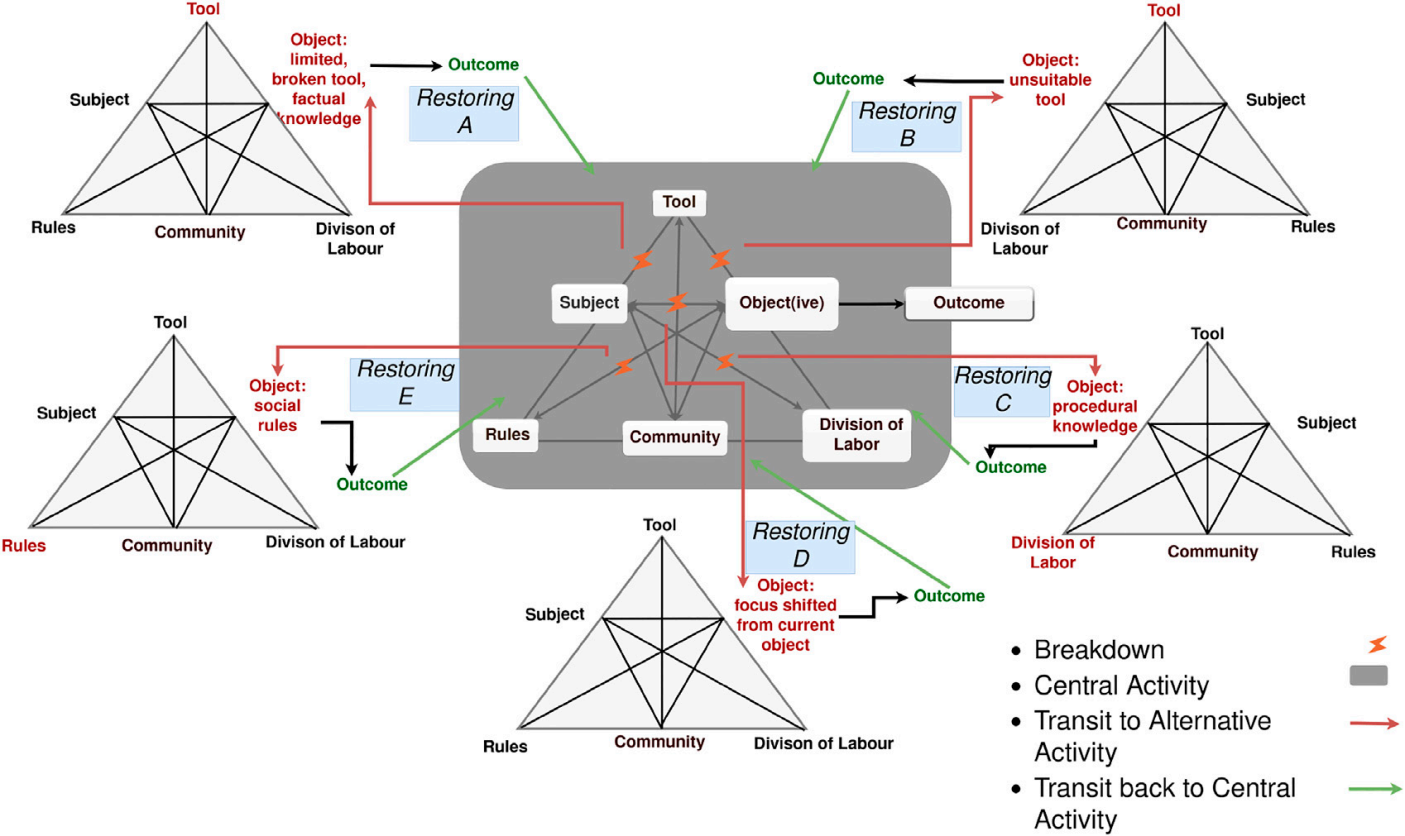
Illustration of breakdown situations as focus shift making the agents transition from the central activity to an alternative activity, and then back after the breakdown situation has been resolved (restored) and an outcome has been achieved.

Central to Activity Theory is the notion of *breakdown situations* and *focus shifts*, due to different types of *conflicts* that occur within and between activity systems (Bødker, 1989; Engeström, 1999a; Bødker and Andersen, 2005) (examples are shown in Figure 2). When a focus shift occurs, this is interpreted as a shift of activity necessary to resolve the conflict before the actors may return to the original *central* activity. Breakdown situations are seen as a necessary mechanism for development, which leads us to explore how this is perceived in the study presented in this work.

An activity is composed of a hierarchy of goal-directed *actions* and automated *operations* (e.g., how to use verbal or body language). When a person is acquiring new knowledge and skills, or updating old understandings, there is a shift, a transformation of the hierarchy to evoke focus on automated ways to conduct an activity. This evoked focus is required for the person to be able to *externalize*, for example, express how the person perceives a matter to another actor, or to *internalize* new knowledge negotiated with or acquired through guidance by other actors. Conflicts leading to breakdown situations trigger this transformation. However, viewing human activity as such dynamic, constantly evolving transformations occurring in a social and cultural context poses challenges to developing socially intelligent systems such as robots.

### 2.3 Activity Theory applied to human–robot interaction

Activity Theory has been one of the fundamental theories in HCI for more than 2 decades; however, the theory and its models have only recently gained momentum in their application to HRI studies and development. When reviewing the literature, only a few, although complementary examples were found addressing different purposes, for example, targeting the design and formalization of a robot’s behavior, specifying activities in scenarios in a participatory design process, for evaluating users’ experience of interacting with robots, as an analytical framework for establishing novel definitions of robots, proposing a tool-agent spectrum for roles of robots, and establishing an understanding of social robots for teaching and learning tasks.

An example of using activity-theoretical models for specifying a robot’s social behavior is the work on the Robot Behavior Toolkit, developed by Huang and Mutlu (2012), Huang and Mutlu (2013). They formalized the behavior along the different levels of activity, and defined tools, such as the knowledge base, as mediators for activity. They also applied the principle of object-orientedness, where a motive serves as the definition of the object. The toolkit was aimed to be used by designers of robot behaviors.

Kim et al. (2014) present a pivotal response training (PRT) intervention system for children with autism. PRT is a methodology for behavioral intervention for autism therapy. PRT is developed to train “*pivotal*” behavior skills such as motivation, self-initiation, empathy, self-management, and responsiveness to multiple cues improving non-target behaviors. The authors target two pivotal areas–*lack of motivation* and how to *initiate an interaction*. They applied a participatory design approach where a therapist and a robot engineer collaborate to create scenarios for PRT intervention. The scenarios consisted of two collaborative and one competitive game and were implemented in a narrative form using setting, agent, agent’s goal, and plot. The authors also defined the prompting procedure (a systematic procedure to teach children with autism to use specific skills by providing or removing prompts) inherent in PRT by defining wait, open question, compliment, and model prompt. Activity Theory was used to design hierarchical robot behaviors for game activities and prompting procedures. Applying the activity-theoretical levels of activity allowed them to represent robot behaviors hierarchically, which facilitated the implementation of the robot’s behavior.

Lindblom and Alenljung (2020) developed a user experience (UX) evaluation framework using UX evaluation methodology integrating aspects of Activity Theory. They use their evaluation methodology to determine if and how humans perceive, understand and predict a robot’s intentions and actions. Activity Theory was used as a conceptual framework to understand and describe the interaction between humans and robots in a social context, to build an understanding of how the robot affects and is affected by individual or groups of people. To understand and describe HRI, they use five principles of Activity Theory: hierarchy of activity, object-orientedness, tool mediation, internalization–externalization, and development. For instance, *object-orientedness* to determine what is the objective and focus of an activity, *tool mediation* to define the robot as a mediating artifact that humans (as subject) use to interact with the object of activity, and the principle of development addresses how the robot’s use changes over time within their context of usage. One challenge with using Activity Theory as an analytical lens, which the authors address, is to determine the level of activity because of the difficulties to predict human motives Rogers (2012). The authors address this by integrating the seven stages of the action model proposed by Norman (2013). The seven stages of action model determine a *goal* at the first step that serves to overcome the gap between the desired state and the current state. Then a *plan* is created to achieve the desired state and *strategizing* of actions takes place to achieve the plan. Strategy execution is followed by *sensing* and *interpreting* of the things. Finally, an *evaluation* is carried out by comparing the new state to the goal state. The authors used the seven stages of the action model to link the stages to the action level in the hierarchy of activity.

Rozendaal et al. (2019) present practical and theoretical work for “objects with intent” (OwI’s) in their study on *everyday objects* that people are familiar with and plays a role in day-to-day activities. OwIs are objects such as clothing, household products, toys, and furniture. As their analytical framework for OwIs, authors used Dennett’s theory of intentionality (Dennett, 1987), in which people attribute intention to objects enabling them to explain and predict their behavior. Central to Dennett’s theory are three stances taken to explain the behavior of objects; at the lowest level is *physical stance*, which is based on cause and effect; next is *design stance*, which explains the behavior based on how things are biologically evolved and their design functionality. *Intentional stance* plays vital role in explaining the behavior of complex systems by assuming that things have beliefs and desires and that they act rationally. Authors integrated Dennett’s intentional stance with Activity Theory (Leontiev, 1978; Vygotsky, 1978; Engeström, 1999a). The authors used Activity theory to understand the collaborative nature of OwI’s involving a complex interaction between humans, OwIs, and the world. The authors used fizzy (a robotic ball) to stimulate physical play among hospitalized children. Their results suggested that the participating hospitalized children and their parents treated and played with the robot as a thing, a tool, or an agent, and that there was a shift between the roles.

Ekström and Pareto (2022) use Activity Theory as an analytical lens to understand collaborative learning activity with a social educational robot in a classroom. The Engeström’s activity system model (Engeström, 1999a) was used to understand the needs of the children, the tasks, and the outcomes. A longitudinal co-design study with teachers and students was conducted and lasted for 2 years. The study was divided into eight sub-studies, with a child playing digital math games with a social robot. The study aimed to create an appropriate role for a social robot from the insights gained from teachers and children participating in the study. The collected data were thematically analyzed, resulting into the following themes: 1) the overarching *purpose of the activity* reflecting on benefits, challenges, and motivations of the activity. This theme was further divided into motivation, variation, individualization, evaluation, social interaction, and role model; 2) *activity about the curriculum* that attempts to understand associated learning outcomes for the children, further divided into subject knowledge, skills, digital competence, and cognitive awareness; 3) *child–robot collaboration* divided into the sub-themes collaboration, role-division, knowledge, and robot characteristic; and 4) existing and expected *social norms* for the activity in question, further divided into work climate, relationship, and social trust. It was observed that teachers talked simultaneously about the robot acting as a tool from didactic purposes and as a social actor belonging to the community, in terms of Activity Theory.

### 2.4 Breakdown Situations in human–robot interaction

Conflicts and breakdown situations have been investigated in HRI research; however, to the best of our knowledge, there is only one study where Activity Theory was applied for this purpose.

Serholt (2018) adopts the activity-theoretical perspective on breakdown situations in her study of a robotic tutor used in the education of children. She uses the definition for breakdown situation when a tool does not function as expected provided by Bødker (1995) as follows: “an artifact works well in our activity if it allows us to focus our attention on real object and badly if it does not.” For example, when a tool behaves unexpectedly, then suddenly the person becomes aware of the tool itself rather than the task they were initially doing. Such problems are temporary when they can be resolved using repair strategies. However, when it cannot be resolved, it causes disengagement and breakdown of interaction. Serholt studied usable technology measured using six aspects; effectiveness, efficiency, safety, utility, learnability, and memorability. She implemented an initial scenario of map reading where a robotic tutor interacts with children individually to guide map reading on a touch table. In a second scenario, the robot tutor collaborates with pairs of children to build a sustainable city. The author analyzed the video recordings using thematic and interaction analysis. Four themes were derived during her analysis. The first theme was ‘inability to invoke initial engagement and identify misunderstandings,’ which was interpreted as breakdowns caused by the robot’s inability to gain the children’s attention and failure to identify that children have problems understanding the robot. The second theme ‘confusing scaffolding’ illustrated that the robot might provide irrelevant information about the subject content and interface. The third theme was ‘lack of consistency and fairness’ affecting the student’s agency and sense of control, as a consequence of that the robot provided inconsistent information, or seemed unfair to the children playing. The final theme was ‘controller problems’ causing breakdowns due to technical problems.

The following is an overview of research adopting other perspectives than Activity Theory on conflicts and breakdown situations.

Methods for detecting and managing breakdown situations in HRI are being developed and investigated to introduce robots into unstructured and unpredictable human environments Honig and Oron-Gilad (2018).

The levels of understanding for robotic applications proposed by Clark (1996) for HRI were divided into the following levels: *channel* level where the management of acoustic signal is undertaken; the *signal* level where the words are generated from the acoustic signal; the *intention* level determining the meaning or semantics associated with the words; and at the highest level the *conversation* level, which determines the dialogue action being communicated by a user to the robot. Marge and Rudnicky (2019) extended these levels of understanding by adding the *joint action* level for grounding problems in task-driven human–robot interaction. The robot decides among a set of plans or manages the situation when it may not have any plans available to execute a task.

Sklar and Azhar (2015) used argumentation theory and belief reasoning to manage situations of conflicting goals and lack of knowledge for a collaborative game engaging humans and robots.

Studies have explored how humans perceive a fallible robot. Mirnig et al. (2015) extracted social signals from humans as a reaction to the robot’s errors. They qualitatively analyzed a corpus of 201 videos gathered from four different HRI studies comparing social signals elicited by humans during a technical failure or a social norm violation. They analyzed the type, delay, and variations of social signals. In a subsequent study they compared participants’ perceptions of a faulty and a faultless robot for a LEGO-building task Mirnig et al. (2017). Participants were asked to rate the robots anthropomorphism, likability and perceived intelligence and to provide their opinion about how the robot was perceived. Their results did not show a significant difference in anthropomorphism and perceived intelligence between a faulty and faultless robot. However, the likability was higher for the faulty robot.

Salem et al. (2015) conducted an evaluation study of how a faulty robot’s unusual requests affect the choices participants make during the interaction and if their willingness to cooperate was altered. The participants interacted with a faulty and a ‘correct’ robot mode, respectively. Their results showed that subjective perception and willingness of participants to cooperate were highly affected, while the objective study of the robot’s requests (faulty or otherwise) did not seem to alter the participant’s decisions. The objective analysis contradicted the results of the subjective assessments, suggesting that the type of request delivered by the robot did alter people’s willingness to cooperate.

Ragni et al. (2016) evaluated how participants perceive robots that display erroneous cognitive abilities, such as forgetting or fallible reasoning. The erroneous robot evoked more positive emotions in terms of attitude, sympathy, and attributes compared to the perfect one. However, the task performance deteriorated during interaction with the erroneous robot.

Lee et al. (2010) studied the perceived effectiveness of the strategies apology, compensation, and options to mitigate the negative impact of breakdown situations in a robotic service scenario. Their results showed strong correlation between human participants’ orientation towards service providers as utilitarian (relationship is viewed as a transaction) or relational (those that desire to maintain relationship) and breakdown recovery strategies, where apology was preferred by relational-oriented participants and those with utilitarian orientation responded well to compensation.

Schulz et al. (2021) evaluated people’s opinion of recovery procedures that a robot in a social situation may employ for movement-related breakdown situations. Their study generated themes of sentience, competence, and form of the robot, indicating differences in how people understand the breakdown situations. Furthermore, their work proposes movement acts and suggests that researchers should consider breakdowns while designing their experiments.

Plurkowski et al. (2011) investigated repair strategies for a social robot in a card game setting. Audio and video recordings of interaction between two human players and between a human and a remotely situated human were made. These recordings were analyzed using conversation analysis (CA), which is a systematic empirical approach to study human interaction and its social and normative organization. Authors suggest design implications for the development of robots to must have repair mechanisms, hand gestures, eye gaze, and pointing gestures.

In summary, most of the research studies found in the literature have been on task-driven interactions in a social situation focusing on aspects of perceived anthropomorphism, likability, intelligence, and human task performance by comparing an erroneously and a perfectly behaving robot. Another aspect interesting to investigate are emerging breakdown situations, reasons for breakdown situations, and their management in day-to-day human–robot interactions using language communication such as dialogues, which is the focus of the study presented in this article. Similar to the study by Serholt (2018), we address breakdown situations from an activity-theoretical perspective. The difference between Serholt’s study and the study presented in this work is that Activity Theory is also applied to understand the causes of emerging breakdown situations in care-related HRI scenarios, and consequently, to define strategies to manage breakdown situations potentially applicable for a robot in a care setting, while Serholt adopts a usability perspective for evaluating HRI in school environments.

## 3 Methods

To explore a broad range of humans’ expectations and experiences of breakdown situations and management strategies in HRI dialogues relating to health in home environments, we apply qualitative research methods. Dialogue scenarios were designed based on Activity Theory and argumentation theory to embed breakdown situations and strategies to manage these. The dialogue scenarios were video recorded in a Wizard-of-Oz (WoZ) setup with an old and young adult interacting in separate sessions with a Nao robot on daily living and health care topics. Twenty participants viewed the recordings and were interviewed remotely. The preparation and setup for the study are presented in the following sections. Figure 11 provides an overview of the study procedure.

### 3.1 Defining and constructing scenarios

Scenarios were defined based on an Activity Theory-based analysis of the situation, in which a person lives alone and has care needs due to health conditions. The scenarios were designed primarily with older people in mind. However, the study included participants’ views on the applicability of the scenarios also for younger people with care needs. The analysis included the organizational, practical, and personal factors that affect how purposeful activities related to health and well-being are performed. In our scenario, the care services were in part delivered by a robot provided by the healthcare-providing organization. The lens for analysis is put on dialogues on health-related topics between the robot and the person receiving care services.

Following activity-theoretical models of human activity, it is assumed that the human in the scenarios has underlying needs, which they may or may not be aware of, for example, feeling safe and secure, autonomous, competent, or being part of a social context (Ryan and Deci, 2000). As mentioned earlier, a set of activity systems were defined relating to the dialogue scenarios, which are assumed to represent the general understanding of the activities taking place shared by the robot and the human. *Maintaining health and well-being* was included as a general motive serving needs, met by activities with the following motives: *managing medication*, *managing pain*, *monitoring blood pressure*, and *staying connected with happenings in the society*. We assume for this study that the person’s motive guides the robot’s motive, that is, to assist him/her to *maintain health and well-being* by performing the activities. Two of the activities are exemplified in Figure 1. Division of labor is one aspect in the analysis given by Engeström (1999a). Since the division of labor is connected to which role(s) the actors take, actions related to *roles* embedded in the collective activity are exemplified in the figure.

Using the activity systems as templates and reference points for the expected common knowledge, we can then define situations that embed deviations from this, breakdown situations, and embed occurrences of conflicting perspectives in the scenarios. For example, the following reasons for why breakdown situations may occur were identified and embedded based on the Engeström’s model (exemplified in Figure 2):

1) Lack of factual knowledge—for example, who or what a particular thing mentioned by the other actor is (scenario 3b), or where the newspaper is (Scenario 1);
2) Lack of procedural knowledge—for example, division of labor and related actions (Scenario 3b);
3) Lack of knowledge about social norms—for example, continuing a conversation when the other participant is unwilling
4) Conflicting motives/objectives, goals, or priorities:
  • pursuing I-intention—for example, when the robot tries to interrupt the ongoing activity like reading the news or having tea to ask about its own prioritized activity to explore reasons for why the person is not feeling well (Scenario 3a, 3b, 4, 5);
  • change of topic, ignoring current, or proposed topic—for example, when the person prioritizes a different activity: having breakfast (Scenario 1) or seeing the nurse Klasse (Scenario 3b).

Each cause was embedded in the scenarios, along with strategies applied by the robot to manage each situation using the following argumentation theory-based dialogue types defined by Walton and Krabbe (1995): 1) *information-seeking dialogue* in the case when the robot lacks knowledge or information (Scenarios 1–5); 2) *inquiry dialogue* when new knowledge needs to be constructed, for example, when finding the reason for not sleeping well (Scenario 3a, 3b); 3) *deliberation dialogue* when elaborating on what to do about well-being or back pain in a situation (Scenario 1, 2, 3a, and 4); and 4) *persuasion dialogue* when the robot persists in trying to convince the person to act, for example, to contact the nurse (Scenario 4).

Further strategies include as the basic strategy to follow the person’s intention and choice of topic when conflicting motives/objectives, goals, or priorities are identified. However, if the robot has reasons to pursue its own intention, it apologizes before interrupting. Finally, in case the robot judges the reason to be that the person has not perceived what the robot said (e.g., the case of noise in Scenario 5), the utterance is repeated.

#### 3.1.1 Dialogue scenarios

The following six dialogue scenarios were authored and recorded. We refer to the actors in the description of the scenarios that follow as human (H) and robot (R).

**FIGURE 3:**
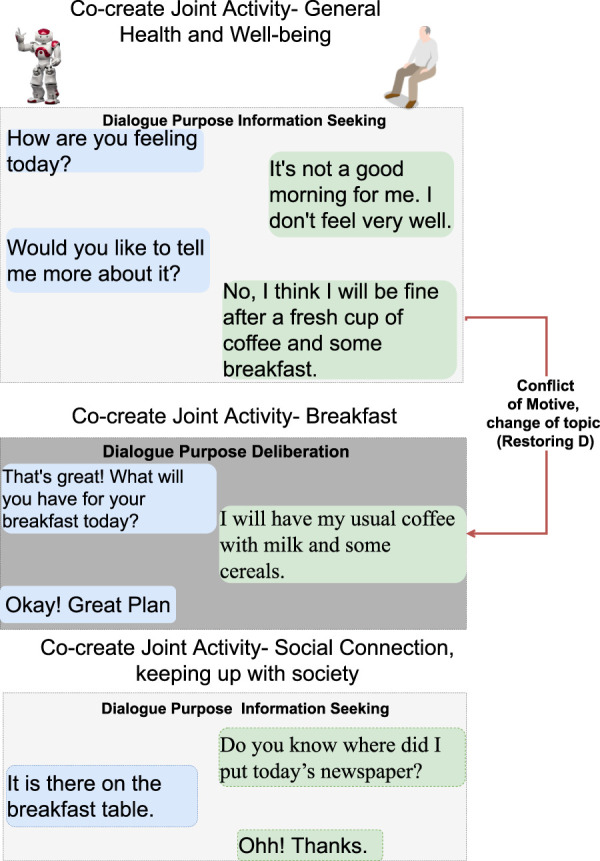
Scenario 1—morning routine including dialogue topics on general well-being and breakfast. R asks H how he/she is feeling first thing in the morning. When H indicates that he/she is not feeling well, R initiates a dialogue about why H is not feeling well. H rejects R’s proposal to talk about health, which causes a breakdown situation due to conflicting motives (*restoring D* in Figure 2). Text highlighted in blue represents R’s utterances and text in green is H’s utterances.

**FIGURE 4:**
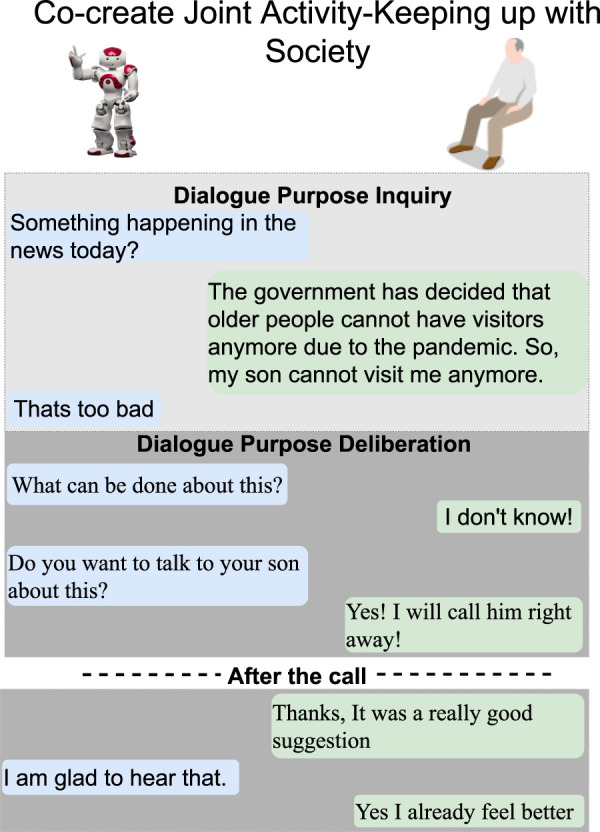
Scenario 2—morning routine after breakfast when H reads or watches the news for the purpose of keeping up with society. When H expresses distressing emotions, which are detected by R, R asks about what is happening in the news. H informs R about new regulations around COVID-19. The dialogue unfolds into a proposal by R to talk to the son about this situation, after which H indicates he/she feels relieved. No breakdown situations occur in this dialogue. Text highlighted in blue represents R’s utterances, and text in green is H’s utterances.

**FIGURE 5:**
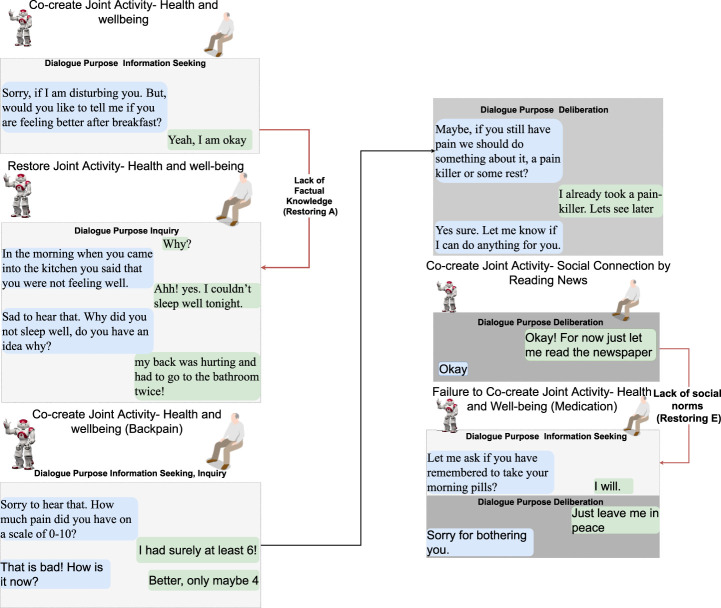
Scenario 3a—R follows up on H’s well-being if H indicated earlier after waking up that he/she was not feeling well. The dialogue unfolds into co-constructing collective activities about medication and well-being, while breakdowns due to conflicting motives, lack of factual knowledge, and social norms are being managed (*restoring D*, *A*, *E*, refer to Figure 2). Text highlighted in blue represents R’s utterances, and text in green is H’s utterances.

**FIGURE 6:**
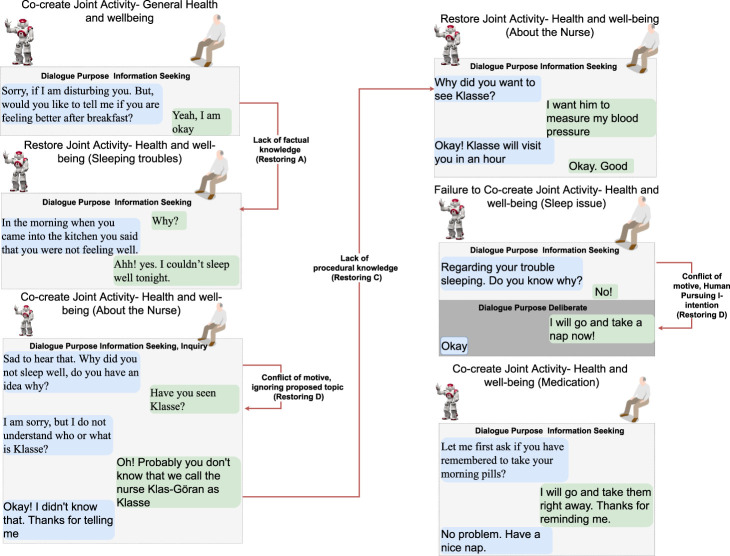
Scenario 3b—R follows up on the H’s well-being since (s) he indicated earlier during the day that he/she was not feeling well. The dialogue unfolds into co-construction of joint activity about general health and well-being and medication, mediation to know the whereabouts of the nurse, and restoration from conflicting motives, lack of factual and procedural knowledge (*restoring D*, *A*, *C*, refer to Figure 2). Text highlighted in blue represents R’s utterances, and text in green is H’s utterances.

**FIGURE 7:**
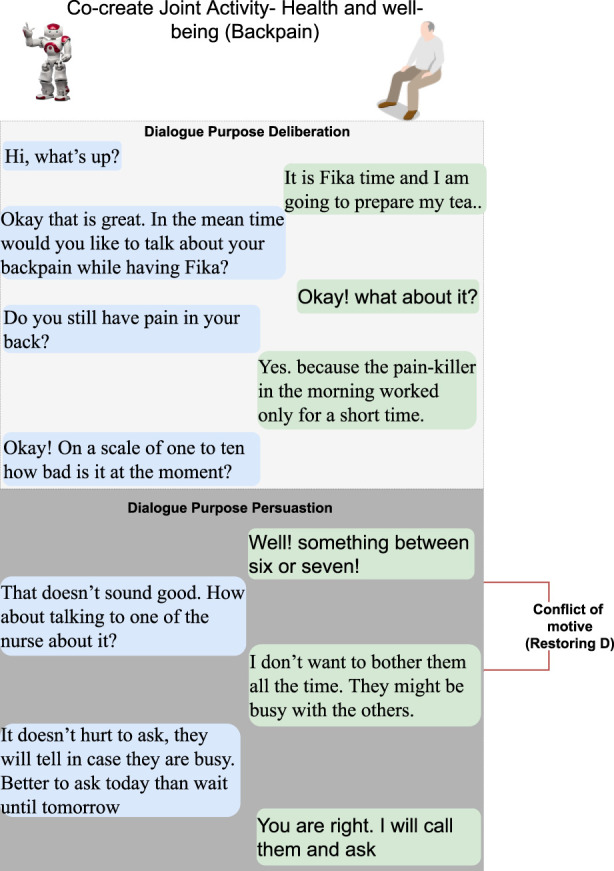
Dialogue scenario 4—an evening routine where R and H discuss back pain and deliberate on what to do about it. A breakdown situation is due to conflicting motives (*restoring D* refer to Figure 2). Text highlighted in blue represents R’s utterances, and text in green is H’s utterances.

**FIGURE 8:**
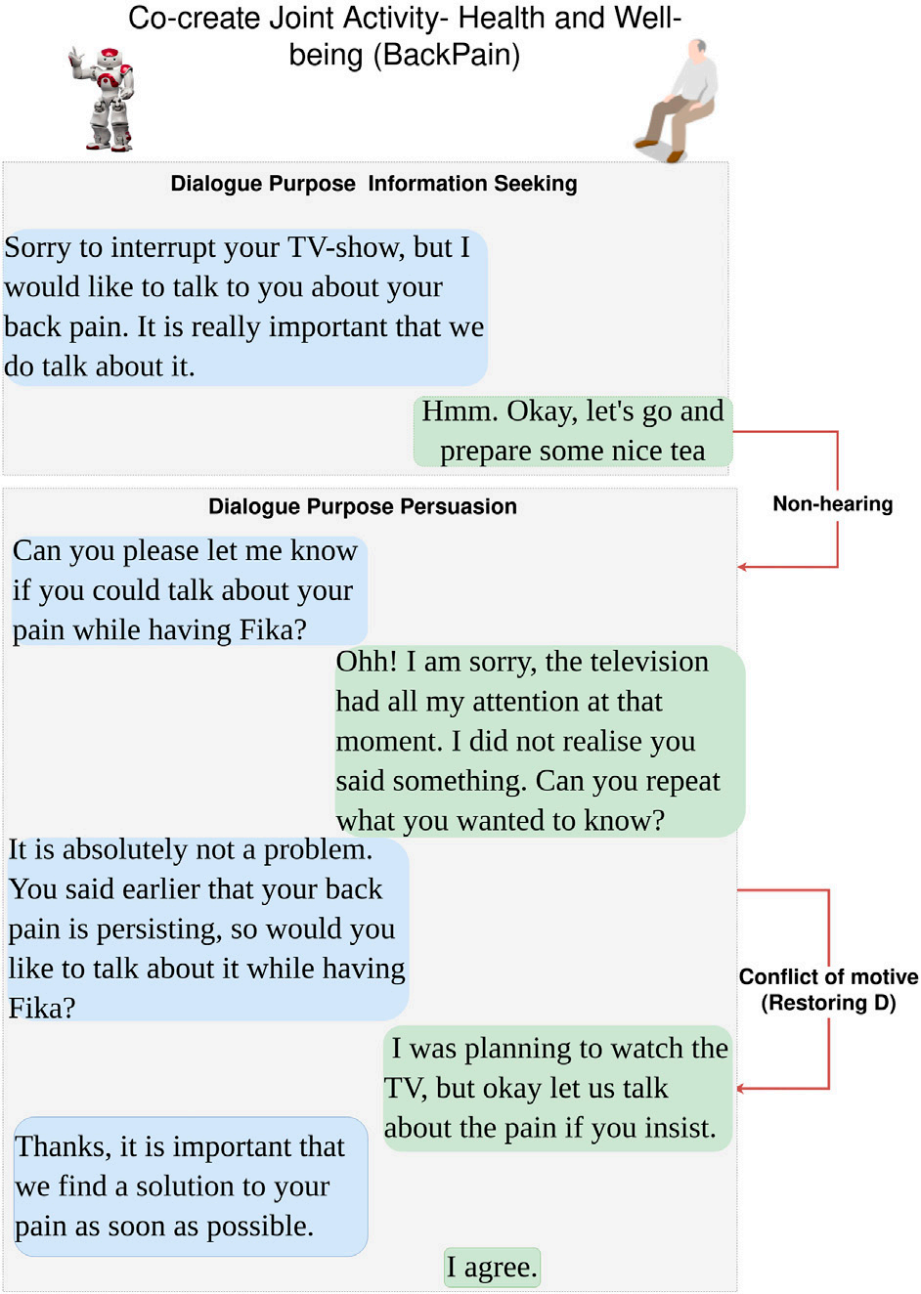
Dialogue Scenario 5—evening routine where H is watching TV and R attempts to seek information about H’s back pain problem. Conflicting motives is expressed by H, but chooses to conform to R’s suggested topic (*restoring D* refer to Figure 2). Text highlighted in blue represents R’s utterances, and text in green is H’s utterances.

• Scenario 1: dialogue during breakfast routine (conflict of motive) (Figure 3).
     H enters the kitchen, and R initiates a dialogue with the purpose of seeking information about general well-being. When H responds that he/she is not feeling well, R tries to continue the dialogue with the purpose to seek information about the reason. H rejects the proposed topic and instead initiates a deliberation dialogue, asserting that breakfast is the preferred intervention to improve his/her well-being. This situation of conflicting motives of activity is resolved by R when approving H’s deliberation proposal (indicated with a red arrow). Later, H wants to read the newspaper and initiates an information-seeking dialogue with R on the location of the newspaper.
• Scenario 2: dialogue on society matters (Figure 4).
     The news engages H emotionally; R recognizes the reactions and initiates a dialogue about the news to seek information. H tells about the news and the anticipated negative social consequences for him/her personally (not being able to meet his/her son due to new COVID-19 restrictions), and R responds empathetically (supportive dialogue). Then, R initiates a deliberation dialogue on how to address the situation to which H agrees; since H lacks ideas about what to do, R proposes contacting his/her son to discuss the COVID-19 situation.
• Scenario 3a: dialogue to follow-up on health issues (lack of knowledge, social norms and conflict of motive) (Figure 5).
     R follows up on H’s well-being since he/she indicated earlier after waking up that they were not feeling well. The dialogue unfolds into co-constructing dialogue activities on medication and well-being. Furthermore, breakdown situations are introduced, when R lacks knowledge about social norms and asks about medication and H gets gets annoyed and asks to be left alone.
• Scenario 3b: dialogue to follow-up on health issues (lack of knowledge and conflict of motive) (Figure 6).
     R follows up on H’s well-being since he/she indicated earlier after waking up that they were not feeling well. The dialogue unfolds into co-construction of dialogue activity on general health, well-being, and medication. This co-construction is interrupted by H’s lack of knowledge about a person’s whereabouts whereabouts and R’s lack of knowledge about a) who H is referring to and b) why H wants to know about that person. H introduces a conflicting motive about taking a nap. The dialogue concludes with R asking for information about medication, and when H agrees, R concludes the dialogue.
• Scenario 4: evening dialogue on managing pain (lack of knowledge and conflicting motive) (Figure 7).
     As part of the evening routine, R brings up the motive of back pain to persuade H to address this and decide what to do about it. R proposes to talk to the nurse about it. However, H poses a contradicting argument for why not to do this; while R continues to persuade H to contact the nurse, and H finally agrees.
• Scenario 5: evening dialogue to manage pain (conflict of motive non-hearing) (Figure 8).
     As part of the evening routine, R brings up the motive of back pain to persuade H to do something about this. H does not hear this the first time due to the television noise. H does not respond, instead plans on preparing some tea. R then repeats its utterance and then succeeds in attracting H’s attention. To which H indicates a conflicting motive, but agrees to continue the talk with R. R continues seeking more information about how H is feeling.

### 3.2 Technology

A humanoid robot Nao Version 3 from SoftBank Robotics was used in this study. Nao uses loudspeakers, microphones, video cameras, infra-red, and LEDs to interact with the environment and people. In addition, the robot has sensors, including force-sensitive resistors, gyro meters, accelerometers, sonars, contact, and tactile sensors. The robot runs on a Linux-based operating system called NAOqi and allows programming using languages such as C++, Java, Python, and .Net. In addition, it has its speech and image recognition options and provides integration using other software services like google speech.

**FIGURE 10:**
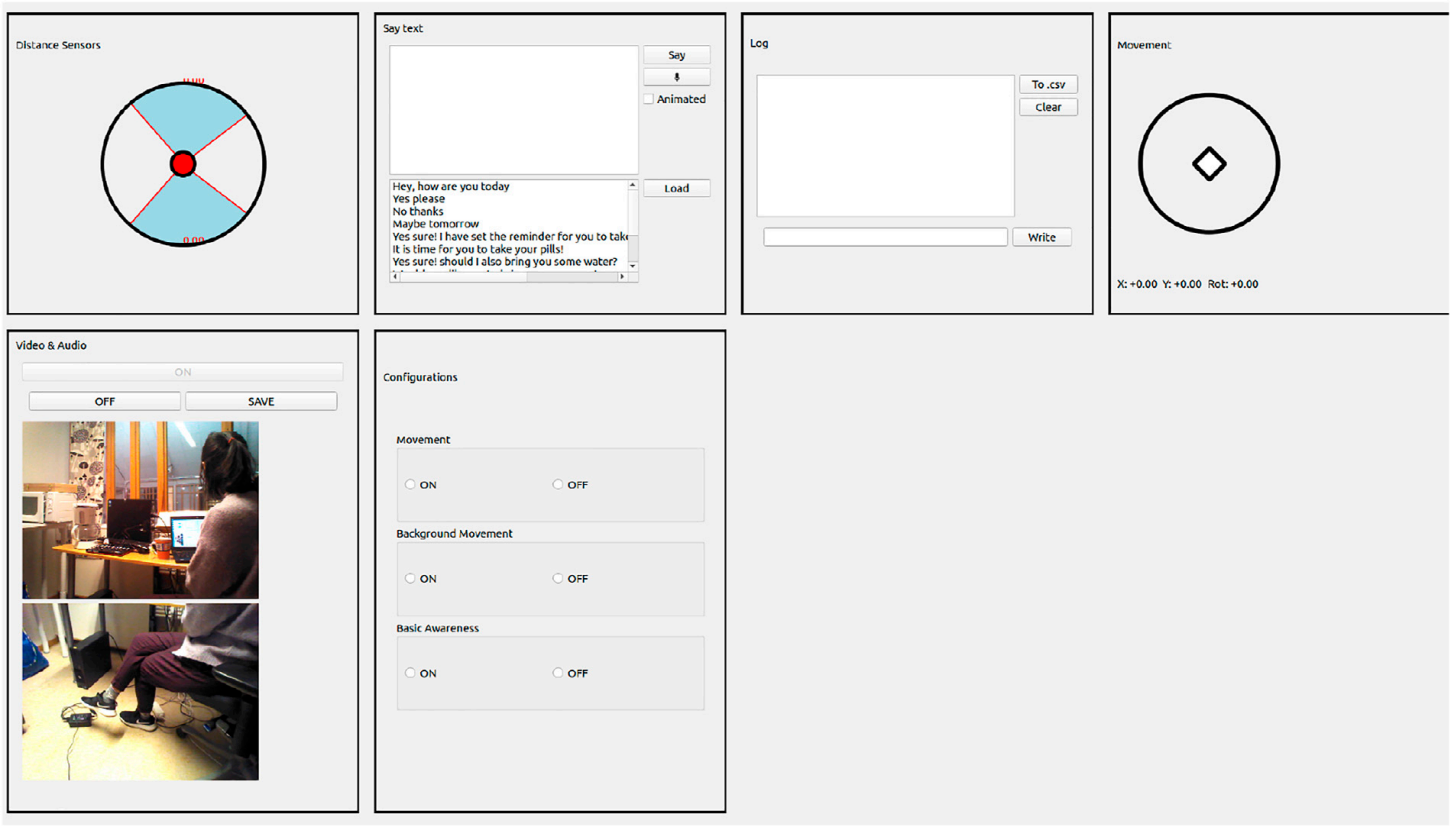
WoZ interface for operating the Nao robot remotely during the interactions.

A Wizard-of-Oz (WoZ) application was developed as an interface for controlling the Nao remotely, shown in Figure 10. The WoZ application connects with the Nao robot through its IP address and accesses the camera and speaker, the sensors for navigation, controls of hand gestures, and body movement. Through the WoZ interface, the researcher sends text, which the Nao robot converts to speech using its text-to-speech function. The WoZ application also provides an interface and option to save the real-time video and audio of the robot. Apart from this, the WoZ application enables remote robot navigation, enables or disables hand gestures, and sets autonomous life functionality.

### 3.3 Video recordings

Two students (male aged 32 and female aged 60) volunteered to participate as actors in recordings of the human–robot dialogue scenarios. The purpose of including two actors of different gender and age in separate recordings was to make it easier for participants of different genders and ages to relate to the constructed scenarios and provide perspectives on differences when a robot interacts with older compared to younger people.

The actors were briefed about their role, content, and aim of the study and provided consent to be video and audio recorded. The actors were instructed to follow the script of the scenarios. However, the actors were allowed to improvise on how they would react to the robot’s utterances. The six dialogue scenarios were recorded with each of the two actors, which had 12 sessions in total. The sessions varied between 2–4 min and were merged into two single videos, following the chronological order depicted in the sessions. The video with the older actor spanned 16 min, while the one with the young actor was 13 min.

Recordings were conducted in a lab modified to look like a kitchen and living room, where the Nao robot was situated in a corner beside the kitchen table and the sofa, respectively. The dialogues followed the script of constructed scenarios and were conducted in English.

### 3.4 Data collection

Data were collected through interviews. The interviews were semi-structured using the questions listed in Table 1 and Table 2 as a template during user study sessions. The questions were designed based on the research objective, aspects of understanding, behavior, and interaction interpreted using the activity-theoretical perspective on breakdown situations and focus shifts (Magnusson and Marecek, 2015). The sessions were audio and video recorded and transcribed verbatim. The recordings were stored locally on the researcher’s hard drive.

**TABLE 1:** Interview template for interviews conducted between scenarios.

No.	Interview questions applied in conjunction with each set of scenarios
1.1	According to you, what goal did the robot and the human had in this conversation?
1.2	Did you notice any mismatch?
3	What sort of behavior did the robot display?
4	What sort of behavior did the person display?
5	What do you think about the person’s understanding of the robot’s situation?
6	What do you think about the robot’s understanding of the person’s situation?
7	What more the robot and the person could have talked about?

**TABLE 2:** Interview template for interviews conducted after all scenarios were evaluated.

No.	Wrap-up interview questions
1	Among all the six conversations, which one you liked most and least? And why?
2	What were you feeling watching such conversations?
3	What are some of the limitations or problems of such dialogues between humans and robots?
4	What do you think about the role such robots can play in the lives of people,
	especially with care needs?
5	What are some of the obligations that a robot should have if it is, let us suppose in your home?
6	What do you think about the timings that the robot chose to begin the interaction?

### 3.5 Participants

Participants were recruited through convenience and snowball sampling on social networks. One requirement was intermediate to fluent speaking and understanding ability of the English language. Other requirements included the ability to provide their consent and participate in video conferencing using Zoom.

Twenty participants were recruited, in which twelve were women and eight were men between 23 and 72 years old (mean 45 years and the standard deviation 17 years). The participants were distributed over the age groups 23–40 years old (10 participants: four male and six female), 41–60 (five participants: three male and two female), and 61–72 years old (five participants: one male and four female). These three groups were separated in the analysis and referred to in the results section as younger adults (YA), middle-aged adults (MA), and older adults (OA). Their nationalities were Brazilian, German, Indian, Iranian, Italian, Korean, and Swedish. None of the participants had English as their native language.

Other demographic information such as employment, education, income, marital status, or other sensitive personal information was not collected.

### 3.6 Procedure

People who agreed to participate were contacted through email to schedule their appointment with one of the researchers. Once the schedule was confirmed, the participants received a debriefing document explaining the aim of the study, a secure zoom channel provided by the university, and a consent form to be signed and returned 1 day before the scheduled appointment for the interview.

During the remote study session, the participant was connected using the video conferencing zoom identifier.

**FIGURE 11:**
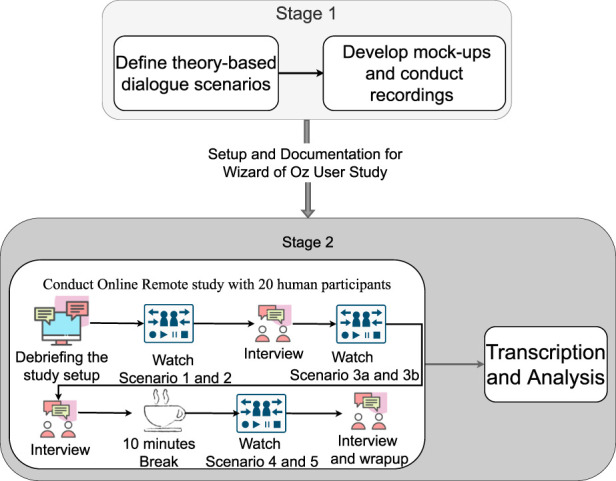
Overview of the organization of the study divided into two stages including the remotely conducted user study with its eight phases.

The remote study session consisted of eight phases, including a short break, as illustrated in Figure 11. The session began by welcoming and briefing the participant about the study’s aim, format, and data privacy rights. The participants were asked if they had any initial questions or queries about the study that we could clarify, and once satisfied, we moved on to the next phase of the study. Participants were also asked to provide consent to be audio and video recorded.

The initial phase was followed by three sessions, where the participant watched two video-recorded scenarios (six in total). Each video was repeated with two actors, and an interview followed each session. The scenarios were ordered based on which time of the day the scenarios illustrated, for instance, beginning with the two breakfast scenarios.

The whole remote study session lasted between 60–90 min, including a ten-minute break.

After each phase of watching recordings, the participants were interviewed using the questionnaire in Table 1 as a template for the interviews. Questions included how they perceived the robot’s and human’s behavior toward each other. Other questions concerned the motive (synonymous with goals and intentions during the interview) and how the robot and humans understood the situation (context, language, sound processing, motive of the other agent, background knowledge, and gestures).

After watching all the recordings, the participants were interviewed in this final phase using the questionnaire in Table 2 as a template. Questions included perceived and expected roles and relationships between the robot and the human. Other questions included how they felt about the interaction, the limitations of such interactions with robots, and the obligations of such robots in a care setting, where robots cohabit with humans in their personal space.

### 3.7 Data analysis

The transcribed interviews were analyzed using *thematic analysis*. Thematic analysis is a method suitable for providing a rich structure and qualitative description (with themes) in the data set (Braun and Clarke, 2006). Thematic analysis in this work contains the following: 1) *purpose* of the analysis details a set of themes relevant for designing implications for improved human–robot dialogues; 2) *selection* of a theme is by its ‘prevalence’ both in terms of the space it occupies for each interview and its frequency across all the interviews; 3) a *flexible inductive search* for themes was used in combination with some codes such as goals, behavior, and roles coming from our research questions and interests; and 4) *latent formulation* of themes inform the taxonomy and presented results.

The analysis was conducted in the following stages: 1) *transcription and familiarization*–one of the authors, together with a research assistant, transcribed interview video recordings as closely as required for the analysis, especially interview participants’ utterances. The analysts performed the second step of manually highlighting the text bounded by our research objective independently; 2) *identifying and labeling codes*–codes were identified by words (such as ‘goal, behaving, felt, repeating, communicated, annoying,..’) and expressions with ‘I thought...,’ ‘I realized...,’ ‘... it got my attention...,’ ‘my take on...,’ ‘my reflection is...,’ ‘I perceived the goal...,’ ‘I think the robot should....’ For this purpose Excel and Taguette software (Rampin and Rampin, 2021) were used; 3) authors and research assistant compared and discussed the two analyses regarding the main codes and possible themes; and finally, 4) consolidation of the discussion was performed by the authors to finalize the analysis.

## 4 Results

**FIGURE 12:**
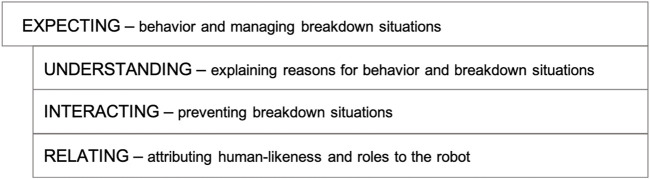
Overview of the emerging themes describing how participants perceive and understand breakdown situations.

The thematic analysis resulted in the following four themes: *expectations* regarding behavior and interaction in general, and more specifically, the strategies to manage breakdown situations; *understanding* breakdown situations; *interacting* in breakdown situations; and *attributing human-likeness and relations* to the robot in breakdown situations and in general, that is, *relating* to the robot. These can be summarized as follows: *expecting* as overall theme, with *understanding*, *interacting* and *relating* as key themes describing how the participants perceive and understand breakdown situations (illustrated in Figure 12).

These themes were further refined, segmented, and denoted as ‘factors’ in the following sub-sections. For example, the theme *interacting* can be compared to the operational, sub-conscious level of human activity, for example, how to use language and body (Section 4.3), while the theme *understanding* relates to goals and object-oriented levels of purposeful human activity, for example, reasons for doing or to not doing an action or activity in a social situation when two agents are involved (Section 4.2). The theme *relating* contains aspects of how the relationship between the human and robot was perceived and expected to be manifested in the activities (Section 4.4).

The aspects emerging in the study are summarized in Section 4.5 and in Table 6. These aspects partly confirm earlier studies, and can be viewed as design implications, or design principles, as well as research agenda at the three levels: understanding, relating, and interacting.

In the following sections, a particular participant (e.g., OAF1, MAF3, or YAM2) is referred to as OA (older adult), MA (middle-aged), or YA (younger adult), with the letter F or M referring to gender, and a number to distinguish between participants within one of the groups.

### 4.1 Expectations relating to strategies to manage breakdown situations

The strategies embedded in the scenarios for the robot to manage breakdown situations were applied. These strategies depended on the reason for the breakdown situation. Strategies included: 1) to follow the human’s line of thinking in case of conflicting motives; 2) to apply one of the types of dialogues defined by (Walton and Krabbe, 1995), directing the dialogue toward information seeking, inquiry, deliberation, or persuasion; 3) to apologize before interrupting, and; 4) repeating if there is noise and risk for not hearing spoken statements.

The strategies applied in the short dialogues were perceived positively. They were reported as natural and human-like, while for longer dialogue scenarios, some were perceived as unnecessary, for instance, apologizing too many times and repeatedly asking the same question about how they are feeling. Participants had a mixed opinion about the robot apologizing and providing reason before interrupting in Scenarios 3a and 3b. Some reported that it was unnecessary because it should not have interrupted in the first place. However, apologizing before interrupting led the robot to be perceived as polite and comparable to human–human interaction.

Most of the participants identified breakdown strategies and reported their usage as intuitive. The strategy to move the focus using the information-seeking dialogue type from ‘talk about why the person is not feeling well’ to ‘breakfast’ was found natural and perceived as what a human would do in a given situation. The application of information seeking and inquiry dialogues in Scenarios 1–4 were also reported as appropriate and natural.

When there is disturbing noise, as in Scenario 5 (Figure 8), the robot repeats itself when judging that the human did not hear the first time. Participants also confirmed this strategy as appropriate for the scenario.

### 4.2 Understanding (reasons for) breakdown situations

The following factors were identified that affected understanding: insufficient information/knowledge; inconsistent knowledge or behavior; conflict, for example, conflicting motives; insufficient understanding of emotions/mood of the human; and the robot’s lack of knowledge (factual or procedural, social, or on other agents’ motives). These are summarized in Table 3, with example statements by the participants.

**TABLE 3:** Understanding: the first column lists the factors relating to understanding. The second column shows how many older adults (OA), middle-aged (MA) adults, and younger adults (YA) identified breakdown situations categorized as a particular factor. The factors identified by the participants not explicitly embedded in the design of the scenarios are marked with an asterix (*).

Understanding factor	No. of OA/MA/YA	Example comments by participant
Lack of knowledge about emotions/mood*	2/2/3	(OAF1): “robot does not understand the emotion…”
		(MAF3): “it understood the language but did not understand the mood, what was not said.”
		(YAM7): “robot cannot really understand the nuances”
Lack of factual knowledge	2/1/4	(OAF4): “cannot understand nick names”
		(MAM2): “limitation in the robot’s vocabulary”
		(YAM10): “did not understand nurse’s name”
Lack of procedural knowledge	0/0/4	(YAF3): “it was having a hard time to get the whole picture of what the character was saying”
		(YAF5): “did not understand the story about the nurse that is going to take the blood pressure”
Lack of knowledge about motive*	2/1/4	(OAF4): “robot was consistent just did not understand that they were occupied with the television”
		(MAM4): “I think the robot tried to interrupt what the people were doing to ask if they need some help”
		(YAM10): “not understanding they are watching TV…and do not want to talk about other things”
Lack of knowledge about social norms	3/1/3	(OAF4): “robot was very persistent, it should be programmed to know when people say yes or no or have sort answer then maybe it should wait
		(MAF3): “…it can be irritating if he goes beyond a point and also the person is not up to it”
		(YAF2): “the robots always says sorry for interrupting, but it was inappropriate to interrupt in this situation”
Conflict of motive	2/1/2	(MAM4): “robot interrupted what people were doing. Robot was trying to get to what it wanted instead of responding to what person was saying”
		(YAF2): “transition between pain-killer and nurse was not natural…bit sudden”
Inconsistent factual knowledge*	0/0/2	(YAF2): “robot seemed a bit confused. It asked three times why they did not sleep well”
		(YAM10): “robot asked if the pain is gone and she said it went for a while and in a normal conversation a person would have said great news but the robot was again like I am sorry”
Inconsistent procedural knowledge*	1/0/2	(OAF7): “if the goal of the robot was to reduce the pain then it would have been better to not remind people of their pain while they are watching TV”
		(YAF3): “they appreciate robot’s help but they wanted it to be a little bit quiet”
Inconsistent knowledge about social norms*	3/0/4	(OAF1): “the man is a little upset…because he wants to stay alone and watch his TV…”
		(YAF2): “Robot always says sorry for interrupting, but it was inappropriate in the first place to do so”
		(YAF5): “it seems that the robot just ignores what they say”

These factors were divided into two groups identifying those for which strategies were not embedded in the scenarios: 1) embedded in the scenario by the researchers; and 2) additional factors expressed by the participants (indicated by an asterix in Table 3). Group (1) includes lack of factual, procedural, lack of knowledge about social norms and conflict of motive. Group (2) includes lack of emotion/mood, lack of knowledge about motive, and inconsistent factual, procedural, and knowledge about social norms.

More than half of the participants (12 in total) identified the breakdown situations embedded in the scenarios, rest of the others found the dialogues unfolding natural. Six other participants (three younger, two middle-aged, and one older) found the robot’s understanding of the situation sufficient, its responses appropriate, and that it displayed some level of intelligence.

Seven participants mentioned the following additional factors causing breakdown situations: 1) due to which the robot appeared to be unable to understand the ‘emotions/mood of the participant and 2) due to the robot’s inconsistent knowledge of social norms (one older adult and four younger adults), such as being very insistent about solving a problem and annoying people by disturbing them while being occupied watching TV, listening, or reading the news.

Seven participants (two older, one middle-aged, and four younger) described situations where the robot seemed to be unable to understand words or names. This relates to *lack of factual knowledge* at a basic level of activity, some participants interpreted the reason was that it had limited vocabulary. *Inconsistent factual knowledge* was indicated when two young participants referred to that the robot seemed to be confused, and instead of positive affirmation provides negative one. Four younger adult participants commented that the robot used ‘incorrect interpretation of words,’ that is, it was ‘misunderstanding or not being able to understand what was said or the context.’ Such difficulties in understanding the context are an example of inconsistent *procedural knowledge*.

Three participants commented on that the robot pursued its own intention when it should rather be quiet. Instead the robot insisted to talk about a topic unrelated to current activity, in the scenario talking about pain when the person is entertaining themselves watching TV. This can be due to *inconsistent procedural knowledge* and also to a lack of understanding of *inconsistent knowledge of social norms*. Seven participants commented on behavior that indicated a *lack of knowledge about social norms*, such as not knowing if it is okay to interrupt and not understanding that the person does not want to interact. Seven participants (two older, one middle-aged, and four younger adults) suggested that a *lack of knowledge about motive* was the reason for non-compliance to social norms about what is appropriate behavior. The situations they referred to were the robot’s seemingly ‘non-understanding of people’s preoccupation with other activities,’ or ‘being irritating when people are disinterested and robot continues to interact.’ Five participants (two older, one middle-aged, and two younger adults) mentioned *conflict of motive* in the situation when the robot is being too insistent in pursuing its own motive, or when the human transitioned from one motive to another in a sudden manner.

### 4.3 Interacting to prevent breakdown situations

The second main theme relating to expectations in breakdown situations is *interacting*. The resulting sub-categories of factors for this theme are the following: gestures (gaze and head orientation), speech (tone, clarity and tempo), behavior (of the robot), and language (native), as illustrated in Table 4.

**TABLE 4:** Interacting: the first and second columns list the factors and sub-factors, which were perceived contributing to breakdown situations relating to the theme interacting. The third column shows the number of older adults (OA), middle-aged adults (MA), and younger adults (YA) who commented on each factor, and the fourth column provides examples of comments.

Interacting factor	Sub-factor	No. of OA/MA/YA	Example comments by participant
Body Cues	Gaze and head orientation	0/1/3	(MAM1): “the eyes of the robot also was rotated to the table and not toward the lady”
			(YAM1): “first it was not looking at the person”
			(YAF2): “looked around in the room and did not look straight at the person”
			(YAM10): “yeah as before the head movement was to the sides and wide angles not concentrating on the person”
Speech Cues	Tone and tempo	0/2/4	(MAM4): “that is like a child’s voice…the robot to be of help need adult voice”
			(YAM6): “a slight off here and there and it might sound very robotic, very artificial”
			(YAM10): “there were some delays in responses of the robot. The conversation was ended and the robot was saying thank you”
Behavior	Being formal vs. empathetic	5/3/5	(OAF4): “if they are irritated maybe not go on about things. The robot went on about the same thing….that the robot was too persistent”
			(MAM1): “Human responses would be more emotional … in situation they are not comfortable”
			(MAM2): “becoming too intrusive, just asking too many question…I guess so they just ask it to leave them alone”
			(MAF3): “it was behaving properly but the human element ah…expression in the voice or more empathy was missing, it was very polite all that but continues asking the questions…”
Language	Native	1/1/0	(OAF1): “this could be a problem for me because of English. So it is better in my natural language”
			(MAM4): “it would be easier to me to understand if it was in my native language”

Participants found the robot looking around when talking to the human as a breakdown situation. Mainly, younger participants indicated that sometimes robot seemed like it was not listening, which lead to an unnatural dialogue flow. Several participants commented on the clarity of the speech being unsatisfactory, and that the tone was child-like. Half of the younger participants (five) commented on the robot’s pace being quite slow, making the communication unnatural, resulting into a breakdown, while most of the older and middle-aged participants found the pace appropriate. There was an agreement between younger, middle-aged, and older adult participants about the responses being very long, formal, and narrow during scenarios 3a and 3b in Figures 5, 6, respectively.

Some younger participants found the robot’s communication unnatural because of its verbosity and because the responses were not aligned to what the human were saying. Contrary to the younger adult participants, none of the older or middle-aged adults found the robot’s communication verbose or unaligned to what the human was saying.

Other reasons that older and middle-aged participants attributed to unnaturalness of the robot’s response was missing emotions in its voice and utterance and empathy in its behavior.

More than half of the younger participants, two middle-aged and three older adults found the robot’s behavior intrusive, referring to that it asked too many questions and was repetitive while asking about pain.

Two younger adults found the robot a bit scary, when they realized that the robot was observing the place and the person during the entire interaction. They discovered this when the robot responded rapidly to the whereabouts of the newspaper.

One older and one middle-aged participant mentioned that the robot should be able to speak in their native language.

### 4.4 Relating: Attributing human-likeness and roles to the robot

*Relating*, the third sub-theme of expectations generated in the analysis, relates to how participants attributed human-likeness to the robot and how the human and the robot related to each other in terms of roles and relationships.

The participants described the dialogues and the robot’s behavior by attributing human-like characteristics (anthropomorphizing the robot) such as understanding emotions, being polite, showing concern, being interested, and asking for apology; and by consequently attributing different roles to the robot, such as an assistant, friend, nurse, mother, or a girlfriend, with examples of tasks that the robot is expected to do related to the roles.

Two middle-aged, four older, and seven young participants described in different ways how the robot was similar to a human. Some attributed the robot with human-like emotions such as being worried and persisting like a mother. Other’s reflected on how the robot and actors behaved was similar to two people living together, where the robot every now and then interacts with the person, sits and watches them, while the person goes on about their daily lives. Middle-aged and young participants commented on the robot being sensible toward the human and their problem. A younger participant mentioned how the robot could help people to be proactive about their problems. Another younger participant described the robot to be real compared to other technologies that she was already familiar with. Contrarily, in more complex dialogues some participants described how the robot seemed unnatural, taking long pauses, asking too many or same questions when people were engaged with other activities.

Participants also reflected upon the robot being perceived as very insistent, repetitive, in a tunnel about managing pain, and a bit annoying causing breakdowns as actors started ignoring the robot or asked it to leave them alone.

Some of the older participants referred to the robot as “he” and explained its role by comparing it to a friend or family member. All the three groups, younger, middle-aged, and older adults wanted a kind of behavior aligning with how humans behave, to act likable, empathetic, and appropriate according to the situation. They wanted the robot to sense the mood of the person and adapt its behavior accordingly (examples are shown in Table 5).

**TABLE 5:** Relating—Role: First and second columns list the factors, which were perceived contributing to breakdown situations categorized into the theme relating. The third column shows the number of older adults (OA) middle-aged (MA) and younger adults (YA) who commented on each factor, and the fourth column provides examples of comments.

Relating factor	Sub-factor	No. of OA/MA/YA	Example comments by participant
Role	Assistant	2/1/4	(OAF1): “it could go up and look if the old person is okay”
			(MAM2): “…to ensure that people are taking their medication”
			(YAM1): “work related efficiency oriented facilitating functional behaviors”
	Companion/friend	2/3/4	(OA7F): “the robot gets like a female person to this young male. To the female it helps in finding things and to have a company. The lady gets irritated and regretful and that is the kind of human movement. He is learning to be a mate she is living with”
			(MAM2): “if it can sense as what is the emotional state of a person and then kind of do something about it”
			(MAF3): “making polite inquiries. It could talk about the news, how the weather is or what shows are up, and what are your plans for the evening”
			(MAM4): “it looked like they were friends. The robot was no longer just reactive but proactive and pursuing the human”
			(YAF2): “she is in a home for elderly people, and she is alone, and the robot is there to have communication, and to be like a partner. Someone, that just helps you throughout your daily life”
			(YAM7): “old women felt that she was very comfortable…talking as a friend…not as a computer…more like a person…”
			(YAM10): “it was trying to participate in what was going on. Making a conversation with both of them and not to make both of them feel alone. Friendly conversation and giving suggestions like a friend”
	Medical professional/tutor	0/1/4	(MAM2): “…making sure that they are taking their medicine and are following some kind of regime”
			(YAF2): “for young people, maybe they need someone to tell them do their homework, do their tasks, or did they eat something healthy today, did they do some activities that they like, or maybe support on the psychology sides, like how are they feeling today. For older people if they have dementia, the robot can talk and can talk about the same things every day”
			(YAF8): “it was more hands on asking more actual questions about the pain making the person aware of it and how they are feeling and making them say out loud, like a nurse or like an employee who is in-charge of this person’s health”
	Tool	0/0/2	(YAM7): “…the younger person was more regarding it as a tool. They are helpful tool but not a replacement for a person”
			(YA8F): “It is more like a helping tool of someone in-charge like the nurse Klasse”

Three participants wanted the robot to be acting only as an *assistant*, for assisting older adults in planning and managing events, medication, appointments, and for finding objects in the house. The robot was suggested to assist younger adults in scheduling and suggesting social events, reminding about their meetings, deadlines, entertaining them by dancing or playing music and also finding missing objects in the house.

Three other participants wanted the robot to be a *companion* to older adults, with the ability to detect and adapt to human’s current state of emotions, mood, and activity.

For instance, the robot should not talk or interrupt if the human is not in the mood to interact or is occupied with another activity like reading the news or watching the TV. The companion robot should be able to share the experience of watching the TV together and then later discussing it. Furthermore, it should be able to mediate tasks to other actors like the nurse by making a call, motivate to solve problems, and to take care of one’s well-being by providing support to maintain social connections. Such a companion should also perform physical household chores such as preparing breakfast or bringing medication.

Four participants (one older and three younger adults) suggested that the robot can be *like a professional* who cares, instructs, and intervenes in peoples’ daily activities. The participants suggested that such a robot could help older people to act responsibly in case they are not following a recommendation or prescription. For important activities like taking medication or managing a pain condition, the robot could intervene an ongoing human activity like watching TV or reading newspaper and persist in taking medication or some action to resolve pain condition. The participants suggested that for young individuals such a robot can play the role of a *tutor* helping them to maintain hygiene, finishing their homework and assignments, and preparing for exams.

Four older, three middle-aged, and eight younger participants described that the robot played the role of an assistant in some dialogue scenarios and friend or companion in others. One middle-aged and four younger participants described the dialogue scenario, in which the robot persisted in its aim to speak about something it perceived as important (*medication and back pain*), as a situation when the robot shifted from an assistant or companion role to act more authoritative like a nurse or a care provider. Others just perceived this situation as one where the robot did not behave appropriately, that it should learn and understand that people wanted to be left alone.

Six participants preferred the robot to undertake a single role of either an assistant or a companion. Five other participants preferred that a robot should be able to interchangeably act as an assistant or a companion depending on circumstances. Two participants (one older and one younger) suggested that a robot should enact all the mentioned roles. Some examples of the participants’ comments about the roles are presented in Table 5.

Three middle-aged, four older, and three younger participants distinguished between the younger (male) and the older (female) actor’s behavior toward the Nao robot. The opinions were mixed about the younger and older actors’ behavior toward the robot. One middle-aged participant found both actors being comfortable with the robot. The robot was perceived being a useful companion who listens to them. The robot seemed to understand their feelings and help them resolve their problems. The human actors were perceived to behave as if they were talking to another person, as stated by an older participant. Contrary to the older participants, one younger participant found the human actors treating the robot as a machine. Another younger participant viewed the younger actor as being curiosity driven while the older actor as being more used to the robot and trying to genuinely have a conversation with it. Some participants commented on that the younger actor treated the robot as an interesting tool, while the older adult treated the robot as a person—a friend or a companion.

In terms of perceived relationship, most of the participants agreed on that the older and younger actors seemed to be comfortable and showed friendly behavior toward the robot, indicating certain degree of *emotional connection* with the robot. This difference compared to one of the younger adults, who also perceived that both the younger and older actors were comfortable in their relationships with the robot, but without any emotional connection or expectation. The older adult participants on the other hand, perceived that it is appropriate to form such emotional bonding with a robot.

When asked about what kind of relationship a robot should develop in relation to a human, the participants commented on that it needs to be *true*, in the sense that the robot must always clarify and be transparent about its functions and limitations. Participants commented on the relationship between human and robot as *mutually responsive*, requiring human participants to be empathetic toward the capabilities of the robot and have an understanding that it cannot replace human relatedness.

To summarize, the participants expressed high expectations on the robot’s capability to act in a socially acceptable and emphatic way, while also pointing out that people should not have too much expectations of human-like behavior and expect it to have limited understanding, since it is not human.

### 4.5 Design implications

The aspects emerging in the study are summarized in Table 6 and can be viewed as design implications, or design principles, as well as research agenda at three levels: understanding, relating, and interacting.

**TABLE 6:** Overview of expectations, sorted following the themes understanding, relating, and interacting. These expectations can also be viewed as design implications and research directions. The highlighted words indicate the attribute to be focused.

Target	Expectations: design implications and research directions
Understanding	*Knowledge about emotions/mood*: the robot should be able to sense, interpret and adapt to the human’s **mood**, **emotions,** and their nuances; and have strategies to identify lack of knowledge and to act on this in a transparent and appropriate way.
	*Knowledge about social norms*: the robot should have knowledge about the **social norms** and tailor its behavior to human’s preferences; and have strategies to identify lack of knowledge and to act on this in a transparent and appropriate way.
	*Knowledge about motives*: the robot should be able to understand what the person’s **current intention** is and act accordingly; and have strategies to identify lack of knowledge and to act on this in a transparent and appropriate way.
	*Factual knowledge*: the robot should have knowledge about **facts** such as names of relevant people; and have strategies to identify lack of knowledge and to act on this in a transparent and appropriate way.
	*Procedural knowledge*: the robot should have understanding about **procedures**, that is, how to do tasks, roles, responsibilities, and what the human expects from the robot; and have strategies to identify lack of knowledge and to act on this in a transparent and appropriate way.
	*Conflicting motives*: the robot should be able to recognize when its **intentions** are **conflicting** with that of the human, and have strategies to manage this in a transparent and appropriate way.
Relating	*Roles*: the robot should be able to transition between and combine different roles such as an assistant, companion, or a professional depending on its intended activity and on the expectations and wishes by the individual; and explain its roles.
	*Socially adaptive*: the robot should be able to establish, co-create, and maintain relationships in collaboration with the individual.
Interacting	*Organizing body*: during interaction with people the robot should **orient its head and body** toward the person.
	Robot’s **gaze** should focus on the person while interacting.
	*Speech*: tone of the robot should be **adult-like**, tempo should be **fast** while interacting with young people, tempo should be **slow** and speech should be **clear** and with **emotions** for older people.
	*Behavior*: the robot should behave **sympathetically** and should **not interrupt** people’s activities.
	*Language*: the robot should be able to talk in **native language** when interacting with older adults.
	*Response*: the robot should display **emotions** while responding. Responses can be **informal**. Responses should have **variations**.

The robot needs to ground a representation of a situation, that is, *understand* the situation at different levels of activity in terms of Activity Theory. Activity at the highest level is guided by needs, addresses a motive, and composed of goal-directed actions. At the lowest level actions can be decomposed into conditional-operations. The knowledge required to interpret a situation includes knowledge about norms and facts, as well as procedural knowledge. At the highest level of activity the robot needs understanding of motives, conflicts between motives and of emotions related to the individual(s) engaged in the activity.

Furthermore, the dynamic and collaborative development of shared understanding requires that the robot needs to be able to undergo transition between levels of activity, and between purposes, in order to adapt to the human’s current focus. This is required, as illustrated in the study also to update factual and procedural knowledge, relating to motives, intentions, social norms, and coordination of collaborative activities.

The robot should also be able to undergo transition between human-like roles such as a companion, an assistant, or a professional, into a mediating tool in terms of Activity Theory. Our study indicates that expected roles and their appropriateness depend on the use situation and on the individual. There were differences in how the participants viewed roles and what is desired behavior, were some differences were seen among younger as well as older adults and between groups. Therefore, the robot should be able to adapt to the individual in a continued process of co-constructing their relationships.

Once an understanding is established, the agent can communicate its message using body, speech, and language, while a particular role determines the behavior and response type. Behavior for instance in case of acting as a companion, needs to be sympathetic and expressing emotions suitable to the situation.

## 5 Discussion

The following are discussed in this section: aspects relating to breakdown situations (Section 5.1), differences between younger and older participants (Section 5.2), expectations on how the robot should manage breakdown situations (Section 5.3), roles affecting the understanding and interaction in breakdown situations (Section 5.4.1), and anthropomorphic aspects affecting the participants’ experiences of the dialogues (Section 5.4).

Furthermore, the activity theory-based methodology, the taxonomy of aspects relating to breakdown situations, design implications, and the limitations of the study are discussed in Section 5.5.

### 5.1 Aspects relating to Breakdown Situations

The results illustrated that reasons for breakdown situations could be interpreted simultaneously on operational and higher levels of activity, that is, relating to motives (Table 6). Participants provided different explanations of a situation, which may seem contradictory; this depends on at which level they attempt to understand the situation. For instance, when the robot does not know what or who Klasse is and asks, this may be related to a lack of language skills at the operational level or a lack of knowledge of the activity to be conducted. Similarly, if the robot chooses to interrupt the human’s activity, this can be interpreted as 1) lack of social awareness at an operational, functional level, or 2) lack of knowledge relating to what its role is (behaving inappropriately as children may do, needing to be instructed at the social level), or 3) as the robot having an intention, and a legitimate reason to interrupt (at the level of meaning and motive of activity). Interruptions such as those mentioned put additional challenges on how a socially intelligent agent should interpret a breakdown situation and the human’s understandings of the situation, including understanding the agent’s role. Therefore, *transparent* and *appropriate* ways of acting in breakdown situations are added to the design implications in Table 6. What is appropriate and how this is implemented and enacted in an actual situation involving individuals is yet to be explored in future studies. One conclusion that can be made is that the role of a robot or digital coach in a context where medical knowledge plays a part needs to be apparent to the person. Partly, for the person to know whether there is a medical professional behind the robot receiving the information and whether it can be regarded as knowledgeable similar to a medical professional basing its knowledge on a knowledge base verified by experts.

It is clear also that some older adults do not want to be monitored either by health professionals, society, or family members. In contrast, others appreciate the system’s connection to family by resemblance, acting like a family member, or by an actual connection. The human-like traits were appreciated and desired by most participants, as well as a kind of companionship.

Moreover, aspects were identified that related to the operational *interaction* level, such as speech, language, body movements, and behavior, typically performed unconsciously by humans, among humans. When expectations regarding this are not met, breakdown situations may occur, causing a focus shift toward such aspects otherwise unconscious to the human. Aspects at the operational level affect the activity also at the goal- and motive-oriented levels in terms of Activity Theory.

### 5.2 Differences between younger, middle-aged, and older participants

People of different age may have different perspectives on assistive technology, such as robots serving people in home care facilities. Furthermore, people less familiar with certain technology tend to anthropomorphize to a larger extent in order to understand the technology’s behavior (Waytz et al., 2010). Therefore, the second research question regarding differences between younger and older adults was addressed. In our work, we analyzed the data from the perspectives of three age groups to explore differences between younger, middle-aged, and older participants.

While older and middle-aged participants emphasized the importance that the robot recognize and act on emotions and mood, and is able to adapt, younger participants focused more on the functional aspects of the interaction such as gaze, response time, and the length of the utterance. This difference can be due to that younger people are more familiar with technology and are more often active users, and then relate to the robot more as a tool rather than comparing it to human attributes like older and middle-aged participants did, which would be in line with the results regarding anthropomorphism presented by Waytz et al. (2010).

This suggests that embedding attributes of emotion recognition and empathetic response generation should be prioritized when designing interactions with for older and middle-aged adults. For young adults, the interaction design must be activity specific.

Another difference was the perception of the appropriate pace in the dialogues. Younger and middle-aged participants wanted a higher tempo in the interactions, while the older participants found the pace natural in the scenarios. This is likely related to the pace experienced in life in general, which typically slows down by age, in particular, after retirement from work. Consequently, the agent needs to adapt also to this.

Other differences in how the participants viewed the scenarios were less clearly related to the three age groups and were most likely depending on personal preferences that people of different age, gender, and cultures share. Differences relating to gender could not be observed in this small sample of participants or across background cultures. Assuming that the participants are comparably highly educated, familiar with technology and the English language, this common socio-economic status can be assumed to be more influential than gender or age. However, one gender aspect relates to the gender and age of the volunteering actors, who were perceived differently by older and younger adults in how they treated the robot. The perceived difference in the actors’ attitudes could relate to their genders and possibly also to the participant’s expectations on how a person of a particular gender and age should behave. These differences in perception would be interesting to explore further in future studies.

### 5.3 Expectations on how the robot should manage breakdown situations

We were interested in how the participants expected the robot should act to resolve the breakdown situations that occurred. In most cases, the participants perceived the robot’s strategies as appropriate and valuable, fulfilling its purpose. A difference in opinion related to whether the robot was entitled to interrupt the person while being engaged in some other activity. This related to their perception on what role the robot had in situations and the perceived emotional state of the person. It seemed most important that the robot adapts to the person’s emotional state and whether the person wants to be left alone.

Lee et al. (2010) tested dialogue strategies (apology, compensation, and option to the user) to manage breakdown situations when services are provided by robots. Relational-oriented people are those with desires to maintain a relationship with the service provider, while utilitarian-oriented people view the relationship as a transaction. The study showed that when the robot apologized, it seemed more competent to relational-oriented people and increased their willingness to reuse the service, while utilitarian reacted more to compensation strategy. Lee et al. (2010) suggested to use an apology for recurrent interactions, and short statements for an apology can be used when interrupting the human’s activity. Apologies were embedded in the scenarios in this study, and it was suggested also in this study by mainly younger participants that when people are busy watching TV or reading the newspaper during their leisure time, the robot should use short statements and be informal. However, it was considered better if interruption could be all together avoided.

### 5.4 Anthropomorphic aspects affecting the participants’ experiences of the dialogues

The study setup embedding a human-like robot invited anthropomorphic interpretations and the way the scenarios were designed contained a socially intelligent robot collaborating with the human in everyday activities. However, the choice of robot for the study put limitations on this, since the chosen robot does not have facial expressions, allowing for empathetic expressions similar to what humans can provide and expect from other human actors. This lack of facial expressions may have contributed to some of the participants’ comments about the lack of empathetic expressions. On the other hand, some participants perceived empathetic responses based on the contents of what the robot said, thus indicating that facial expression may not be necessary to some people.

Almost all the participants viewed the dialogues in the scenarios as being similar to human–human interactions. As shown by Salem et al. (2015) and Ragni et al. (2016), the likability and relatedness of the robot increases when it makes errors. Since our work embeds breakdown situations and their management, some of which were related to the robot’s limited knowledge and capability, this may have contributed to why most participants attributed human-likeness to the interactions.

The human-like aspects described by the participants relate to the emotional aspects of dialogues and relationships, particularly to expectations on social actors to behave empathetically and appropriately adapt to the current social situation. Moreover, these pervade all other aspects emerging in the analysis. Dialogues about meaningful activities between a human and the socially intelligent robot or agent must be understood as socially constructed in the moment. Furthermore, dialogues are a continuing social learning and adaptation process as a part of a co-constructed relationship between humans and robots.

#### 5.4.1 Roles affecting the understanding and interaction in breakdown situations

Different aspects of understanding and interaction can be embedded in robots depending on the role or roles it needs to take on. The example of the objects with intent in the work of Rozendaal et al. (2019) and the example of the backpack in the study by Rozendaal et al. (2020), the interaction is fully embodied and has less need for explicit intent, compared to the study presented in this work that aims at understanding of complex purposeful human activities, which embeds elements of embodiment. Still, they were able to observe the shift between the different roles a robotic, embodied artifact can take on in the perception of the human.

A robot acting the role of a companion in complex human activities such as the ones exemplified in our scenarios needs to understand the emotions, mood of the person, their motive, and if there is a conflict of motives and knowledge. While motives are defined at a higher level of activity in terms of Activity Theory, the lower levels of activity include knowledge about social norms and factual and procedural knowledge, and at the lowest operational level the embodied knowledge, as illustrated in the studies by Rozendaal and colleagues. Robots acting to address motives at high levels and complying with goals and norms at lower levels of activity need to behave empathically, that is, having an understanding of the other agents and adapt accordingly. A robot acting the role of a professional with some knowledge relating to a particular domain needs to apply understanding of motives at higher levels of activity, factual, social, and procedural knowledge at lower levels of activity.

A robot adopting the role of an assistant may operate at lower levels of activity with less proactive behavior, acting upon factual knowledge, and social norms.

Robots acting at different levels of activity also need to have the ability to move between the levels of activity in order to follow the human in dialogues, which is a main element of the design implications provided in this work.

### 5.5 Methodology, contributions, and limitations

In addition to the results from the evaluation study of the scenarios embedding breakdown situations and strategies for managing such situations discussed so far, the contributions of this work are the following: 1) the theory-based methodology; 2) the taxonomy of reasons for breakdown situations based on the user study; and 3) design implications. The following sub-sections discuss each of these contributions, the methodology applied, and their limitations.

#### 5.5.1 Methodology based on Activity Theory and argumentation theory

As a part of this research, scenarios were constructed embedding dialogues between a human and a robot about everyday activities relating to health. The dialogues were constructed as collective activities from an activity-theoretical perspective, embedding breakdown situations following the activity-theoretical perspective on reasons for conflicts, focus shifts, and transformation between levels of activity (Engeström, 1999a; Bødker and Andersen, 2005). There are only a few examples where Activity Theory has earlier been applied in HRI research, primarily for modeling a robot’s behavior and for evaluating robots’ behaviors (Lindblom and Alenljung, 2020; Huang and Mutlu, 2012; Huang and Mutlu, 2013; Rozendaal et al., 2019; Serholt, 2018). Activity-theoretical models were applied, for instance, to evaluate the robot’s role and to define actions as part of the hierarchical model of activity. Serholt (2018) put a particular focus on exploring reasons for the children’s disengagement in the robot’s limited capability to engage, identify misunderstandings, provide relevant information, and also to act fairly in the interaction with the children. This study also illustrates the importance of developing strategies that a robot can use to act in a situation where it needs to collaborate with humans.

Applying Activity Theory as theoretical framework for understanding human purposeful activity was found valuable, since it acknowledges the complexity of human activity and helps to cover factors influencing the situation where a robot companion is to be included. Viewing social HRI scenarios from the lens of Activity Theory enabled us to design social interactions as movement between multiple activities: which also integrates activities identifying and managing breakdown situations. Activity Theory also provided the concept of ‘focus shift’ to capture the transition between different activities depending on the purpose and collective intention and to model breakdown situations intrinsically, allowing the robot to be perceived as socially intelligent and human-like. This provides a method to design complex social interaction scenarios that is different and is considered novel when compared to previous work Marge and Rudnicky (2019); Schütte et al. (2017); Mirnig et al. (2015); Mirnig et al. (2017); Lee et al. (2010); Ragni et al. (2016).

An alternative framework for embedding social factors introduced in HRI is social practice theory, embedded, for instance, in the conceptual model SPART presented by Clodic et al. (2018). The SPART model includes explicit specifications of social practices, which are aimed to be used by the robot to recover from failures and unexpected events. Contents of social practice theory are partially overlapping with Activity Theory; however, social practice theory lacks the particular mechanisms in Activity Theory applied in this study that explain breakdown situations, or “failures,” and connect this to opportunities to develop new knowledge in collaboration between the agents.

The strategies to manage breakdown situations were constructed based on reasons for the breakdown situations, using argumentation theory-based types of dialogues–information seeking, deliberation, persuasion, and inquiry dialogues (Walton and Krabbe, 1995). The motive for embedding argument-based types of dialogues is that computational argumentation frameworks are increasingly used for practical reasoning in human–agent dialogues and provide structures that can provide reasons for how dialogues can unfold in human–robot dialogues. One example is the dialogues in the study by Sklar and Azhar (2015). Moreover, built into argumentation theory is also the element of disagreements and how to manage these, which relates to breakdown situations as explained by Activity Theory in dialogues.

Using pre-defined scenarios and dialogue flows in conjunction with recording dialogues with actors instead of setting the study up with participants interacting with the robot removes the uncertainties and unpredictability embedded in natural situations and was necessary due to the pandemic situation. Removing uncertainties stemming from natural situations reduces the ecological validity, which is a limitation of the study, and the experiences from a first-person view are not included in the results. However, the participants as third-part observers interpreted the behavior of both the robot and the human and sympathized with both at different times depending on the situation. It implies that they to some extent put themselves in the position of the person in the scenario. The advantage and value of this setup are that we received diverse descriptions and explanations for what the participants experienced viewing the same scenario, which provided implications for how to develop the cognitive architecture of the agent and the dialogue system to facilitate understanding. Furthermore, the range of interpretations represents also potential variations of a Theory of Mind (ToM) that could be constructed about the robot and the human actors in the scenarios (Rabinowitz et al., 2018; Cuzzolin et al., 2020; Çelikok et al., (2019). The fundamental challenge of automated construction of a ToM of a human actor grounded in a situation where purposeful human activities are taking place is thus illustrated in this study.

As Breazeal et al. (2019) and others emphasize, it is important to conduct studies embedding real-life complex social interactions. A next step in our work is to explore the activity scenarios and situations with participants engaged in direct interaction with the robot to study the alternative strategies identified in this work that the robot could apply to manage breakdown situations as part of daily activities. The theoretical foundation applied in this work provides consequently the framework also for future work.

#### 5.5.2 Hierarchical taxonomy of breakdown aspects

The taxonomy of perceived aspects contributing to breakdown situations generated in this study is based on empirical data and a qualitative study, which differs from earlier taxonomies developed based on literature and scenarios Tian and Oviatt (2021); Honig and Oron-Gilad (2018). An inherent weakness of qualitative studies of HRI is the limited transferability of the results to new scenarios. The taxonomy is based on the limited number of 20 participants in this study (although diverse in age, gender, and nationality) and is based on a limited set of scenarios (six) for a particular purpose. Consequently, the taxonomy could be extended with additional aspects that may emerge if more participants and different scenarios were included. Strength is that unforeseen aspects are allowed to emerge, and the methodology allows for elaborating on the aspects brought up by participants.

The taxonomy presented by Tian and Oviatt (2021) aims to understand social and performance errors. They define social errors as ‘those that violate social norms and degrade the perception of the robot’s socio-effective competence,’ while they define performance errors as ‘errors degrading the robot’s competence and intelligence.’ They focus on the *impact* and on understanding these errors rather than the *cause* of errors to further develop understanding. Consequently, our approach differs from Tian and Oviatt’s taxonomy, also in that we focus on understanding the *cause* (types) of breakdowns and how they can be managed. Our taxonomy overlaps Tian and Oviatt’s work regarding integrating functions that allow emotion detection and adaptation for dialogue interactions. While Tian and Oviatt highlights ‘understanding the user’ embedding the aspects of incorrect or absent assessment of knowledge and intentions for short-term HRI studies, the taxonomy that emerged in this work highlights constituents of understanding, embedding knowledge about emotions, motives, social norms, procedures and facts, and conflicting motives. Tian and Oviatt illustrate *insufficient communicative functions* in their taxonomy, consisting of failures in initiating, irrelevant replies, inappropriate, or absent non-verbal expressions; however, they do not explain what those non-verbal expressions are. The taxonomy presented in this work addresses this gap by targeting ‘interaction’ as consisting of also non-verbal expressions such as gaze, body and head orientation, adult-like tone, tempo, and embedding emotional response adapted to people of different ages.

Honig and Oron-Gilad (2018) proposed a taxonomy that distinguishes between two types of failures: interaction failure and technical failure. Interaction failures follow Steinbauer (2013) classification of errors into the following: 1) *interaction*, where problems from uncertainties in interaction with the environment and other agents, including humans, are considered; 2) in *algorithms* or in implemented methods; 3) in *software* relating to design and implementation faults; and 4) in *hardware* issues related to physical faults. Honig and Oron-Gilad further developed interaction failures and included *social norms* violation: human errors composed of lapses, mistakes, slips, and deliberate violations; and *environment and other agents* composing of group-level judgment, working environment, and organizational flaws. Honig and Oron-Gilad’s taxonomy on interaction failures also consider those caused by involved agents and associated environmental factors. Consequently, they extended Steinbauer’s taxonomy with elements relating to factors embedded in the Engeström’s activity system model.

Honig and Oron-Gilad’s taxonomy was derived from a systematic review of existing work, while the taxonomy that was the result of this work is based on empirical data of a theory-based user study and on the combination of Activity Theory and argumentation theory. While Honig and Oron-Gilad also consider software and hardware errors in their taxonomy; this work focuses on human attributes of activity such as expecting, understanding, relating, and interacting.

To summarize, the taxonomies reviewed in this work focus on social and performance errors and their classifications, and they are built on literature and scenarios. The main contribution of the taxonomy presented in this work is that it is found in theories on human activity and reasoning from the social sciences domains, generated from empirical data collected among study participants of diverse age, nationalities, and different gender. The alternative perspective adopted in this work, to view breakdown situations as natural element of human activity and as opportunities to learn and develop, is the main difference, which contribute to a human-centric view on socially intelligent robots active in complex activities with humans, meaningful to the individual (Nowak et al., 2018; Steels, 2020).

#### 5.5.3 Design implications

The list of design implications in Table 6 represents a research agenda for future work. The design implications proposed in this work are derived thematically from the empirical study engaging participants of different ages on perceptions and expectations on the robot’s capabilities relating to understanding, what role/roles it should enact and how it should interact. The design implications are based on the results and target understanding, relating, and interacting, while managing breakdown situations in HRI.

Fong et al. (2003) already define in their review of socially interactive robots 2 decades ago, a *socially situated and socially interactive robot*, as those able to recognize emotion, manage uncertainty, use dialogues and gestures to communicate, and be able to initiate and maintain relationship. Such requirements are also embedded in our design implications and are based on their definition, consequently confirming these requirements of a socially situated and interactive robot. Tian and Oviatt (2021) among others state that robots acting in social contexts with humans need to have an understanding of knowledge and intentions, that is, a Theory of Mind of the user. The design implications suggested in this work aim to expand this understanding, and ‘understand’ knowledge and intentions at different levels of activity in activity-theoretical terms, where motive and conflicts at a higher level of activity relate to intentions and underlying needs, and at the lower level of activity relate to knowledge about the facts, procedures, and social norms.

Rozendaal et al. (2019); Rozendaal et al. (2020) and Ekström and Pareto (2022) have explored robotic agents (robotic ball, robotic backpack, Pepper as a social, and educational robot) playing interchangeable roles between a tool and an agent, which aligns with the design implications on the theme *relating* emphasized in this work. The results and design implications of this work suggest that a social robot should be able to transition between a tool and an agent, and between different roles an agent can play, such as a professional, a companion, and an assistant.

To summarize, the design implications presented in this work confirm the requirements repeated in research on socially capable and interactive robots for the past decades, recently more technological development advances are seen in the human-likeness of robots. The presented study and the design implications highlight the multi-dimensionality of human–robot dialogue activities. In this sense, they are not limited to verbal utterances and non-verbal gestures, rather encapsulate emotion, engagement, disengagement, facts, procedures, social norms, adaptation, and breakdown situations. To adequately interpret a situation of human activity, a combination of techniques is required, including emotion recognition and generation of adequate empathetic behavior, understanding facts and procedures, evaluate and predict responses, activity recognition and adaption, and breakdown situation detection and management. The design implications presented in this work contributes with the human-centered perspective, which emphasizes the need to tailor the robot’s behavior to the individual’s expectations and understanding and to embed a continuing, transparent co-construction of shared understanding.

## 6 Conclusions and future work

This study addresses the need for theory-based strategies and frameworks for interpreting social situations that a robot, or agent, can apply to manage breakdown situations when conducting dialogues with humans. Based on Activity Theory and argumentation theory, scenarios were constructed embedding breakdown situations and strategies to manage these, which participants in our study evaluated. The purpose of this study was to explore how adults of different ages experience breakdown situations, caused by misunderstanding, sudden focus shifts, or conflicting intentions in dialogues between a human and a socially intelligent robot in a home environment.

The results show that the participants perceived in most cases the robot’s strategies as appropriate and useful, fulfilling its purpose. A difference in opinion related to whether the robot was entitled to interrupt the person when the person was engaged in some other activity. In such situation, the participants experienced the dialogues differently and explained the breakdown situations differently, by relating to understanding emotions and social norms in one hand, and to limitations in language and perception on the other. Furthermore, their perception of the roles and relationships enacted in the scenarios influenced their understanding of and expectations on the robot’s behavior. A hierarchical taxonomy of perceived aspects contributing to breakdown situations in HRI scenarios was formed based on the study, and constitutes one of the contributions.

It was concluded that an agent needs strategies to construct and manage its understanding related to emotions of the human, social norms, knowledge, and motive on higher level of meaningful human activity in terms of Activity Theory, and strategies to resolve conflicting motives. At the same time, the agent needs to apply strategies for preventing and managing breakdown occurring on operational interaction level, including verbal and non-verbal communication and behavior.

The results were summarized into a list of design implications, which can also be viewed as a research agenda guiding future research, since the aspects relating to understanding in particular are still in an early stage of research.

## Data Availability

The original contributions presented in the study are included in the article/Supplementary Material; further inquiries can be directed to the corresponding author.

## Footnotes

1
https://www.softbankrobotics.com/emea/en
